# CyanoStrainChip: A Novel DNA Microarray Tool for High-Throughput Detection of Environmental Cyanobacteria at the Strain Level

**DOI:** 10.1021/acs.est.3c11096

**Published:** 2024-03-08

**Authors:** Hao-Yue Shu, Liang Zhao, Yanyan Jia, Fei-Fei Liu, Jiang Chen, Chih-Min Chang, Tao Jin, Jian Yang, Wen-Sheng Shu

**Affiliations:** † Guangdong Magigene Biotechnology Co., Ltd., Shenzhen 518081, PR China; ‡ School of Food and Drug, Shenzhen Polytechnic, Shenzhen 518081, PR China; § Institute of Ecological Science, Guangzhou Key Laboratory of Subtropical Biodiversity and Biomonitoring, Guangdong Provincial Key Laboratory of Biotechnology for Plant Development, School of Life Sciences, South China Normal University, Guangzhou 510006, PR China; ∥ School of Ecology, Sun Yat-sen University, Shenzhen 518107, PR China; ⊥ One Health Biotechnology (Suzhou) Co., Ltd., Suzhou 215009, PR China

**Keywords:** cyanobacteria, strain-level detection, microarray, harmful bloom, intraspecific variation

## Abstract

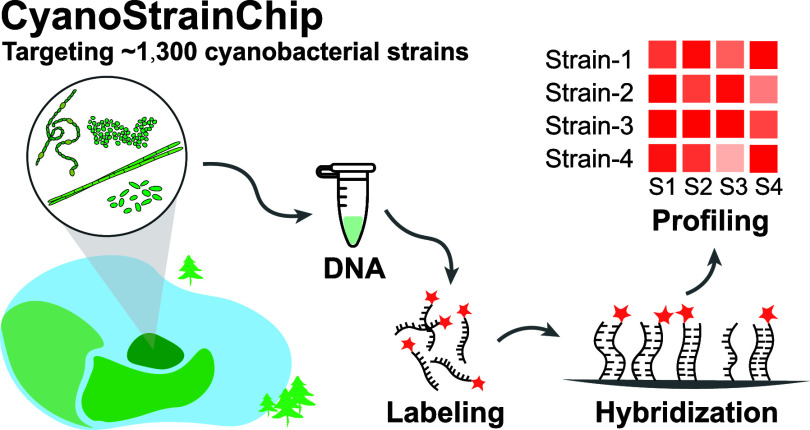

Detecting cyanobacteria in environments is an important concern due to their crucial roles in ecosystems, and they can form blooms with the potential to harm humans and nonhuman entities. However, the most widely used methods for high-throughput detection of environmental cyanobacteria, such as 16S rRNA sequencing, typically provide above-species-level resolution, thereby disregarding intraspecific variation. To address this, we developed a novel DNA microarray tool, termed the CyanoStrainChip, that enables strain-level comprehensive profiling of environmental cyanobacteria. The CyanoStrainChip was designed to target 1277 strains; nearly all major groups of cyanobacteria are included by implementing 43,666 genome-wide, strain-specific probes. It demonstrated strong specificity by *in vitro* mock community experiments. The high correlation (Pearson’s *R* > 0.97) between probe fluorescence intensities and the corresponding DNA amounts (ranging from 1–100 ng) indicated excellent quantitative capability. Consistent cyanobacterial profiles of field samples were observed by both the CyanoStrainChip and next-generation sequencing methods. Furthermore, CyanoStrainChip analysis of surface water samples in Lake Chaohu uncovered a high intraspecific variation of abundance change within the genus *Microcystis* between different severity levels of cyanobacterial blooms, highlighting two toxic *Microcystis* strains that are of critical concern for Lake Chaohu harmful blooms suppression. Overall, these results suggest a potential for CyanoStrainChip as a valuable tool for cyanobacterial ecological research and harmful bloom monitoring to supplement existing techniques.

## Abstract 2

Existing methods can hardly achieve both high-throughput and high-resolution in detecting environmental cyanobacteria. This study develops a novel microarray detection tool for targeting over a thousand cyanobacterial strains.

## 1 Introduction

Cyanobacteria represent a vast and ubiquitous phylum of photoautotrophic prokaryotes, characterized by an extraordinary level of genetic diversity.^1^ These microorganisms are presented in nearly all environments and play pivotal roles in ecological systems, serving not only as vital contributors to the Earth’s oxygen atmosphere but also as prolific generators of secondary metabolites.^1^ Under certain circumstances, cyanobacteria can give rise to harmful blooms that release toxins into freshwater and marine environments, posing a serious threat to ecosystem structure and function, as well as to water quality for recreational, potable, fisheries purposes, and human health.^2−4^ Indeed, profiling the cyanobacterial diversity and abundance in diverse ecosystems will be of vital importance for ecological study and harmful algal bloom monitoring, assessment, and management.

However, the intraspecific variation in cyanobacteria has commonly been overlooked in current studies, leading to generalizations formulated at the species level or above, without considering the potential for strain differences.^5^ Many comparison studies against multiple isolates belonging to the same cyanobacterial genus (or species), such as *Raphidiopsis*, *Planktothrix*, and *Microcystis*, have demonstrated a great extent of intraspecific diversity in their morphology, physiology, toxin, and genetics.^6−10^ For instance, previous papers focused on the microcystins producer *Microcystis*, which is a concern for human and animal health, found that there is substantial variability in the synthesis of microcystins among individual strains belonging to the same species.^8,11^ The nutrient availability of cyanobacteria is crucial to understanding how harmful bloom formation is affected by environmental conditions. Studies have shown considerable intraspecific variability in *Raphidiopsis raciborskii* in response to phosphorus-depleted conditions, which can be attributed to the different expressions of phosphorus acquisition genes and inorganic carbon uptake genes.^12−14^ These studies support the notion that identification of environmental cyanobacteria at a broad taxonomic level (species or above-species level) should be taken with caution due to the presence of significant physiological differences among strains of the same species.

Nevertheless, current methods for high-throughput detection of environmental cyanobacteria can hardly reach the strain level. Polymerase Chain Reaction (PCR)-based DNA sequencing targeting marker genes is the most widely used technique to overcome the limitations of using phenotypic characterization in cyanobacterial diversity assessment.^15^ As early as 1997, a set of oligonucleotide primers for the specific amplification of 16S rRNA gene segments from cyanobacteria was developed.^16^ However, these PCR-based sequencing methods are unable to identify taxa at the species level or blow because their resolution power is limited due to DNA fragments being only a few hundred nucleotides long.^17^ Direct metagenomic sequencing of nucleic acids from environmental samples has become a powerful tool for exploring the composition of complex environmental microbial communities in the past ten years. Although strains within communities can be successfully identified by metagenomics through bioinformatic pipelines, such as inStrain^18^ and PStrain,^19^ certain limitations do exist. Given the high complexity of environmental samples, sufficient sequence depth is often required to improve sensitivity and specificity, which also means huge costs of sequencing and computation, which are still unaffordable for many researchers. Moreover, low technical reproducibility and difficulties in accurately quantifying the relative abundances of closely related strains in metagenomic analysis can introduce significant bias.^20,21^ Therefore, a new approach is needed to address these shortcomings of existing methods.

DNA microarrays, which consist of tens of thousands of DNA fragments arrayed on small glass slides, were initially developed for gene expression profiling.^22^ Since then, various types of DNA microarrays have been developed for microbial detection in diverse environments, for both functional and taxonomic profiling.^23−28^ In comparison to sequencing-based methods, microarrays provide several advantages, including high reproducibility, reliable quantification, relative affordability, and ease of use.^20,29^ Given the maturity of high-density microarray technology and the rapid accumulation of cyanobacterial genomic data,^30^ we developed CyanoStrainChip, a DNA microarray tool, for high-throughput and high-resolution taxonomic identification of environmental cyanobacteria. To achieve this, 43,666 genome-wide, strain-specific probes were designed for targeting 1277 cyanobacterial strains. Subsequently, we examined the usefulness and limitations of this tool in detecting cyanobacteria using *in vitro* mock communities and field samples. The resulting CyanoStrainChip has proven to be capable of accurately detecting cyanobacteria in complex environments at the strain level.

## 2 Materials and Methods

### 2.1 Custom Microarray Design

A total of ∼2500 cyanobacterial genomes were used for designing strain-specific probes, which included 163 genomes previously sequenced in our earlier publications,^30,31^ and ∼2300 genomes obtained from the NCBI GenBank database. To reduce data set redundancy, we employed FastANI to generate whole-genome similarity metrics and all genomes were clustered into strains at 99% similarity threshold.^32−34^ For each strain cluster, one representative genome was retained for the downstream analysis. In total, 1277 genomes remained, comprising 458 genomes from pure cultures, 556 single amplified genomes, and 263 metagenome-assembled genomes. Further details on the remaining genomes can be found in Table S1.

We employed a *k*-mer-based method to discover strain-specific oligonucleotides, as described by Tu et al., with minor modifications.^35^ Detailed information on screening strain-specific oligonucleotides as described in the Supporting Information. We further filtered candidate probes based on various oligonucleotide properties, including probe hybridization free energy, melting temperature, probe secondary structure, specificity, and complexity, using criteria described previously.^36^ For each strain, 30–40 specific probes were selected if enough probes remained; otherwise, all remaining probes were kept. The selected probes were then subjected to Agilent eArray 5.0 program for microarray customizing (Custom CGH, 8 × 64K platform; Agilent, Santa Clara, CA).

### 2.2 Mock Community Preparation

For the specificity assessment of CyanoStrainChip, 23 cyanobacterial pure cultures from 13 genera (Table S2) were obtained from the Freshwater Algae Culture Collection at the Institute of Hydrobiology (FACHB, Wuhan, China). The pure cultures were cultivated in BG11 medium at 25 °C with a light-dark cycle of 12:12 h under a light intensity of 30 μM photons m^–2^ s^–1^. The genomic DNAs were prepared by the CTAB method for downstream analysis. Eight mock communities, differing in composition, were constructed with three replicates by mixing isolate gDNAs.

Two pure cultures (*Microcystis aeruginosa* FACHB-928 and *Nostoc* sp. PCC 7120) were used to assess the sensitivity and quantitative capability of CyanoStrainChip. For each isolation, a serial dilution of gDNAs (ranging from 0.05–100 ng) were subjected to CyanoStrainChip analysis in triplicate.

### 2.3 Field Sample Collection and Preparation

Twelve water samples were collected at three stations (CH1 (N31°41′, E117°24′), CH2 (N31°34′, E117°23′), and CH3 (N31°37′, E117°45′)) in Lake Chaohu (Anhui, China) during the bloom season on September 23rd, 2020. The water samples were labeled as “Low,” “Medium,” or “High” based on their chlorophyll concentration of 17.7, 74.1, and 144.2 μg/L, respectively. Ten liters of surface water were collected at every site from a depth of 0.5 m. The water samples were filtered through 0.22 μm polycarbonate filters to collect all aquatic microorganisms, which were frozen at −80 °C for further experiments. Total DNA was extracted from the frozen samples using a Power Soil DNA Isolation Kit (Biomiga, USA) for CyanoStrainChip and 16S rRNA sequencing analysis. The DNA concentration and purity were monitored on 1% agarose gels. 16S rRNA sequencing data are freely available in the NCBI database (Accession: PRJNA1060477)

To compare the taxonomic profiles revealed by CyanoStrainChip and metagenomic sequencing, one cyanobacterial bloom sample collected in Reservoir Dashahe (N 22°52′, E112°42′, Guangdong, China) on November 17, 2019, was prepared for both CyanoStrainChip analysis and short-gun sequencing on Illumina HiSeq2500 platform (Illumina, USA). Metagenomic sequencing data are freely available in the NCBI database (Accession: PRJNA970118).

### 2.4 Microarray Experiments

For each sample, a predetermined amount of synthetic DNA was added to 1 μg of extracted DNA as a spike-in control. Subsequently, the mixed DNA was labeled with Cy-3 (or Cy-5) fluorescent dye (GE Healthcare, Vacaville, CA, USA) using random primers and Klenow fragment of DNA polymerase I. Labeled DNA was then purified using a QIAquick Purification kit (Qiagen, Valencia, CA, USA), and the NanoDrop 8000 UV–vis Spectrophotometer (Thermo Scientific; Waltham, MA) was used to measure the yield and degree of labeling. Each sample was supplemented with a total of 42 μL of buffer containing 1× HI-RPM hybridization buffer, 1× aCGH blocking agent, 0.05 μg/μL of Cot-1 DNA, and 10% formamide. The mixture was then vortexed thoroughly, spun down, and incubated at 95 °C for 3 min, followed by incubation at 37 °C for 30 min. The samples were subsequently hybridized with CyanoStrainChip at 67 °C for 24 h with a rotation at 20 rpm in an Agilent hybridization oven (Agilent Technologies, Inc., Santa Clara, CA, USA). For posthybridization washing, an Agilent Wash Buffer Kit (Agilent, Santa Clara, CA) was used for removing nonhybridized or partially hybridized labeled sample DNA from the array’s surface to minimize signal noise.

### 2.5 Preprocessing of CyanoStrainChip Data

The tiff images of microarrays scanned on Innopsys InnoScan 900 scanners (Innopsys, Carbonne, France) were processed by Agilent Feature Extraction (AFE) software (Agilent, Santa Clara, CA) to generate spot raw intensities and a series of statistical indices. We took several microarray data preprocessing steps to offset systematic variation and filter false-positive detections before downstream analysis (see Figure S1). Briefly, the raw fluorescent signal of each probe was first background-corrected and normalized by using spike-in control signals. Subsequently, probes with nonsignificant signals were removed based on AFE-generated features. In the last step, we used a Probe Detection Rate (PDR) value of 0.75 as a threshold to filter out false-positive detections. Detailed information on data preprocessing was described in the Supporting Information.

### 2.6 Statistical Analysis

To evaluate the effectiveness of strain detection using the CyanoStrainChip, Precision-Recall (PR) and Receiver Operating Characteristic (ROC) curves were computed on the mock community experiments data sets by varying the PDR threshold. ROC and PR curves as well as the areas under the ROC curve (AUROC) and PR curve (AUPR) were calculated using the PRROC (v1.3.1) package in R.^37^ The bar chart, box chart, and scatter diagram were plotted by ggplot2 (version 3.3.3) in R 3.5.1. Linear regression analyses were performed in R version 3.5.1. All statistical comparisons were performed using a nonparametric Mann–Whitney test in R 3.5.1.

## 3 Results and Discussion

### 3.1 Strain-Specific Probe Screening and Array Design

**Figure 1:**
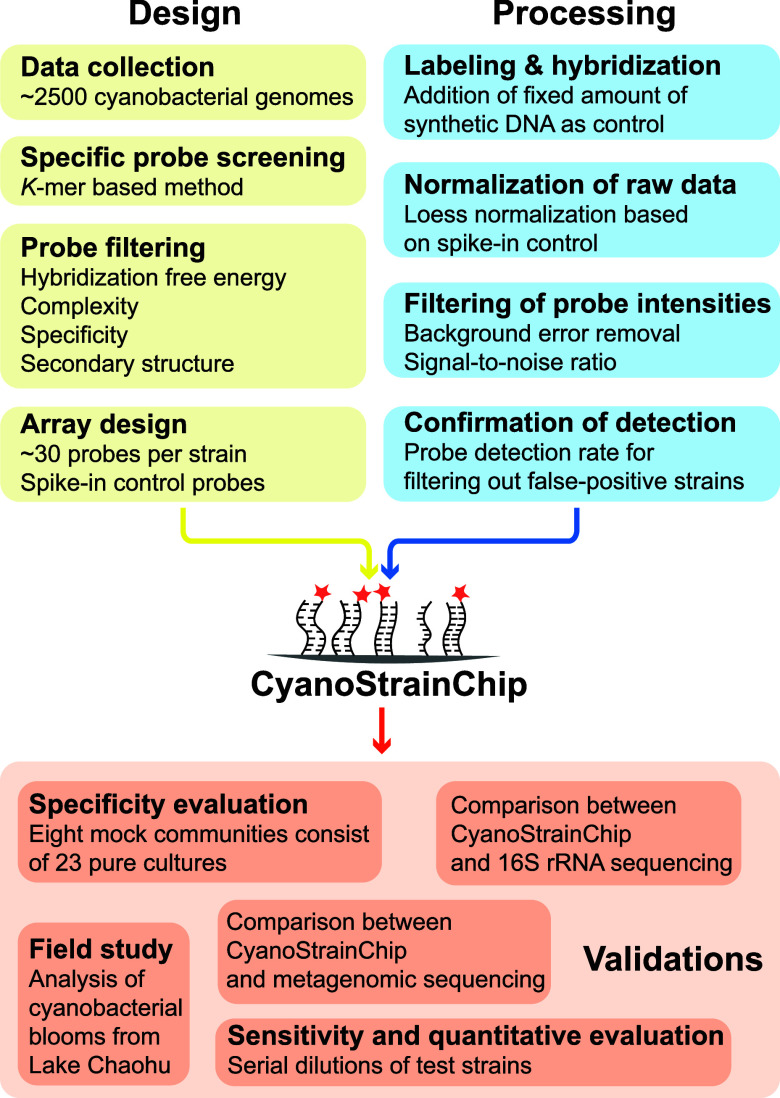
Depiction of the key stages in the development of CyanoStrainChip.

The major steps for the development of CyanoStrainChip are listed in Figure 1. A genome-wide screening of strain-specific probes is crucial to the high-resolution detection capabilities of CyanoStrainChip. To optimize probe hybridization, a suite of criteria was employed for probe selection, including contiguous similarity to nontarget genomes, hybridization free energy, probe secondary structure, GC content, melting temperature, and probe self-folding energy. The oligonucleotide properties of remained probes were computationally evaluated (see Figure S2). Most of the probes displayed less than 20 continuous complementary matches with nontarget strains, GC content between 0.3 to 0.75, hybridization free energy between −85 to −60 kcal/mol, and melting temperatures between 65 to 82 °C. These assessments are consistent with previous probe selection criteria, indicating a reasonable design of probes.^27,36,38^

**Figure 2:**
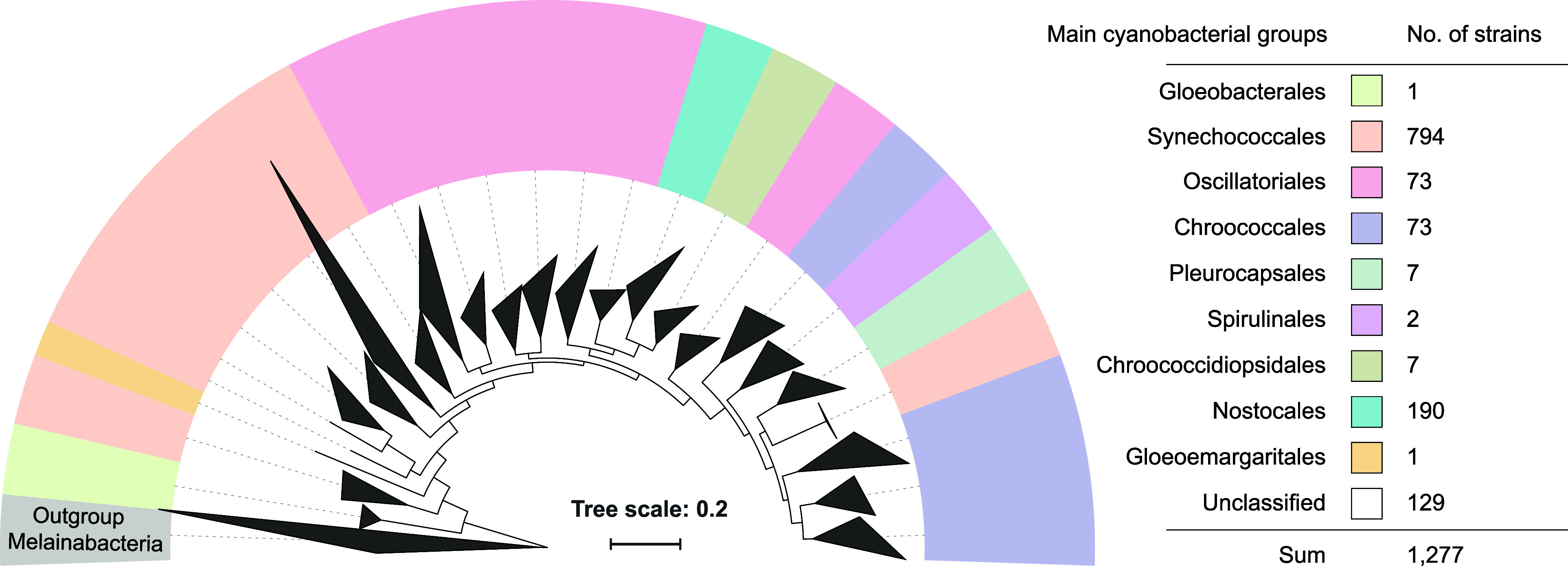
Summary of the target cyanobacterial strains of CyanoStrainChip organized by the main taxonomic groups. Branching relationships of cyanobacterial main groups are based on phylogenetic analyses from previous study, utilizing 834 cyanobacterial-specific benchmarking universal single-copy orthologs.^30^

Despite stringent filtering of candidate probes to minimize cross-hybridization reactions with nontarget strains, there remains a possibility of such events.^29^ To reduce the influence of cross-hybridization effects on the sensitivity and specificity of CyanoStrainChip, the array was designed to include dozens of unique probes per strain. In total, the CyanoStrainChip comprised 43,666 probes targeting 1277 strains belonging to 40 families of nine orders, (refer to Figure 2 and Table S3). Among them, approximately 80% (34,911) were located entirely within gene regions, 6% (2622) spanned both genes and intergenic regions, and 14% (6096) were exclusively located within intergenic regions (Figure S3A). The probes within genes were found to be highly enriched in genes related to COG category M (cell wall/membrane/envelope biogenesis, odds ratio = 2.23, *P* < 0.001), Q (secondary metabolites biosynthesis, transport and catabolism, odds ratio = 1.93, *P* < 0.001), U (intracellular trafficking, secretion, and vesicular transport, odds ratio = 1,79, *P* < 0.001), and W (extracellular structures, odds ratio = 1.86, *P* < 0.001) (refer to Figure S3C). This observation suggests that genes linked to these functions may harbor greater genetic diversity within cyanobacterial taxa, making them crucial for selecting strain-specific probes. Furthermore, we found 423 probes were located within regions corresponding to biosynthetic gene clusters of eight common cyanobacterial toxins (Figure S3B), indicating that the CyanoStrainChip holds the potential to distinguish different genotypes of toxin-producing cyanobacteria.

Another key feature of CyanoStrainChip is the inclusion of spike-in control probes, which were evenly distributed across the array. Conventional normalization methods of microarray data to offset nonbiological differences between samples are based on the assumption of equal target DNA amounts between different samples, however, this assumption is inherently flawed due to variations in target DNA yield in environmental samples.^39^ To improve this, synthetic nucleic acid sequences as spike-in controls were added to each sample at the same amount before fluorescent labeling. Analysis of mock community samples and field samples has shown that this approach is reliable and reproducible, enabling accurate normalization of CyanoStrainChip data (detailed results of the validation of the spike-in controls can be found in Supporting Information).

### 3.2 Specificity Evaluation via Mock Communities

**Figure 3:**
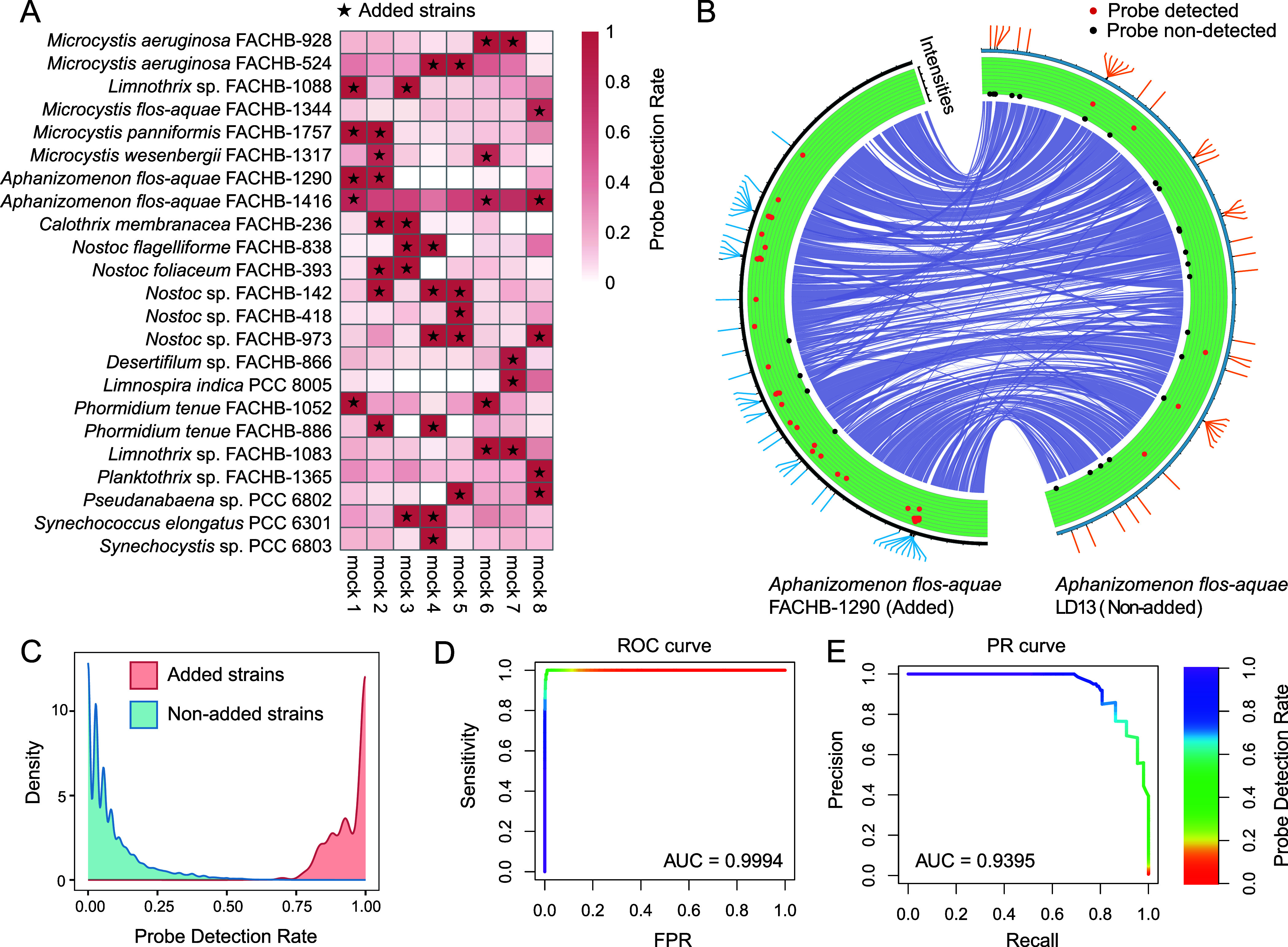
Specificity evaluation via mock communities. (A) Heat map representing the Probe Detection Rate (PDR) of added and nonadded strains across mock communities. (B) Schematic representation of the specific detection of two closely related strains, Circos plot showing conserved synteny between *Aphanizomenon flos-aquae* FACHB-1290 (left) and *Aphanizomenon flos-aquae* LD13 (right), the outer ring represents the location of probes and the inner ring represents the signal intensities of probes. (C) Comparison of density curves of PDR for true-positive detections (added strains) and false-positive detections (nonadded strains). The ROC curve (D) and PR curve (E) of CyanoStrainChip in detecting cyanobacteria by varying the PDR as the detection threshold within the range [0,1].

The specificity of the CyanoStrainChip was evaluated using eight mock communities, composed of a mixture of multiple strains of cyanobacteria, totaling 23 strains across 13 genera (refer to Figure 3A and Table S2). We expected to precisely identify the added strains in each sample while avoiding nonadded strain detections. Initially, we observed that a substantial number of probes associated with nonadded strains exhibited significant signals on the array, suggesting that cross-hybridization events were more prevalent than anticipated. However, it was also noticed that these false-positive probes were likely scattered randomly across the array. Here, we defined a new term, Probe Detection Rate (PDR), which represents the proportion of specific probes that print significant signals for a strain. As shown in Figure 3C, the PDR distribution of added strains and nonadded strains revealed separate density curves, which inspired the realization that the PDR value could be utilized to distinguish between true and false positive detections. As shown in Figure 3A the added strains had an average PDR value of 94.5%, meaning that the majority of strain-specific probes from the added strains showed a significant signal. In contrast, the nonadded strains had an average PDR value of 8.5%. By employing a PDR threshold criterion of 0.75, a near-perfect outcome was observed that all added strains were correctly detected in each combination. One nonadded strain was also identified with a PDR value slightly greater than 0.75 in the mock community 7 and was deemed false positive (Figure 3A). Figure 3B illustrates a schematic representation of the specific detection of two closely related strains. Their probes are scattered throughout the genome in variable regions as depicted. In the *in vitro* experiment, *Aphanizomenon flos-aquae* FACHB-1290 was added to the mock sample and the vast majority of its probes displayed a marked fluorescence intensity, whereas *Aphanizomenon flos-aquae* LD13 was not included and only a minimal number of probes were lighted with a faint signal. We calculated the ROC and PR curves by varying the PDR as the detection threshold within the range [0,1] for the mock community experiment results. The area under the curve (AUC) of the ROC and PR was 0.9994 and 0.9395, respectively (refer to Figure 3D, E), demonstrating successful performance and high recovery rate of the CyanoStrainChip by using PDR as the indicator of detection. Moreover, to substantiate the efficacy of CyanoStrainChip in intricate environments, urban grassland soil DNA was added to three mock community samples as a background and reanalyzed. The cyanobacterial detection outcomes of three types of samples (pure soil DNA, pure strains, and strains with soil DNA) were compared. We found that although more probes printed significant signals in strains with soil DNA samples than pure strains samples, the identified strains in them were completely consistent when using a detection threshold of PDR > 0.75 (Figure S5). These findings indicate the robustness of CyanoStrainChip in the presence of disturbances within intricate environments.

The prevalence of cross-hybridization events in microarray applications for microorganism detection presents a significant challenge, particularly in the identification of closely related microorganisms.^29,40^ This array was designed to minimize the negative effects of cross-hybridization through two key strategies: (1) the screening of strain-specific probes on a genome-wide scale and (2) the implementation of the PDR score for cross-hybridization quality control. Due to the lack of whole-genome data, previous detection microarrays for microorganisms generally relied upon targeting only a small segment of the genome, such as the 16S rRNA gene.^25,41,42^ The detection resolution of these arrays was designed to be above the species level because the majority of microbes belonging to the same genus display a marker sequence similarity of over 95%, thereby rendering the probability of cross-hybridization between closely related species quite high. In this study, the genome-wide, nonconserved sequence was chosen to design specific probes, it offers the best chance of finding probes with low reciprocal similarity between closely related strains.

Despite those probes being in perfect conformity with the *in silico* optimal parameters, we still found that numerous cross-hybridization events persisted in actual microarray experiments. The reason for this phenomenon remains unclear. Several quality control approaches in microarray data processing were reported, which aim to detect and eliminate the potential cross-hybridization events through statistical models.^43−45^ In this study, we proposed that a PDR score greater than 0.75 can serve as a robust indicator for control of cross-hybridization-induced false-positive detections. The assumption is that it is highly unlikely for over 75% of specific probes from one strain to print significant signals due to cross-hybridization simultaneously. This assumption is also supported by a previous study which found the risk of false-positive gene identification on Hydrogenase Chip by cross-hybridization can be minimized by the requirement that 90% or more of the probes targeting a given gene are “bright”.^26^ Overall, the results of this section highlight the accuracy and reliability of CyanoStrainChip in detecting cyanobacteria at the strain level.

### 3.3 Sensitivity Evaluation

**Figure 4:**
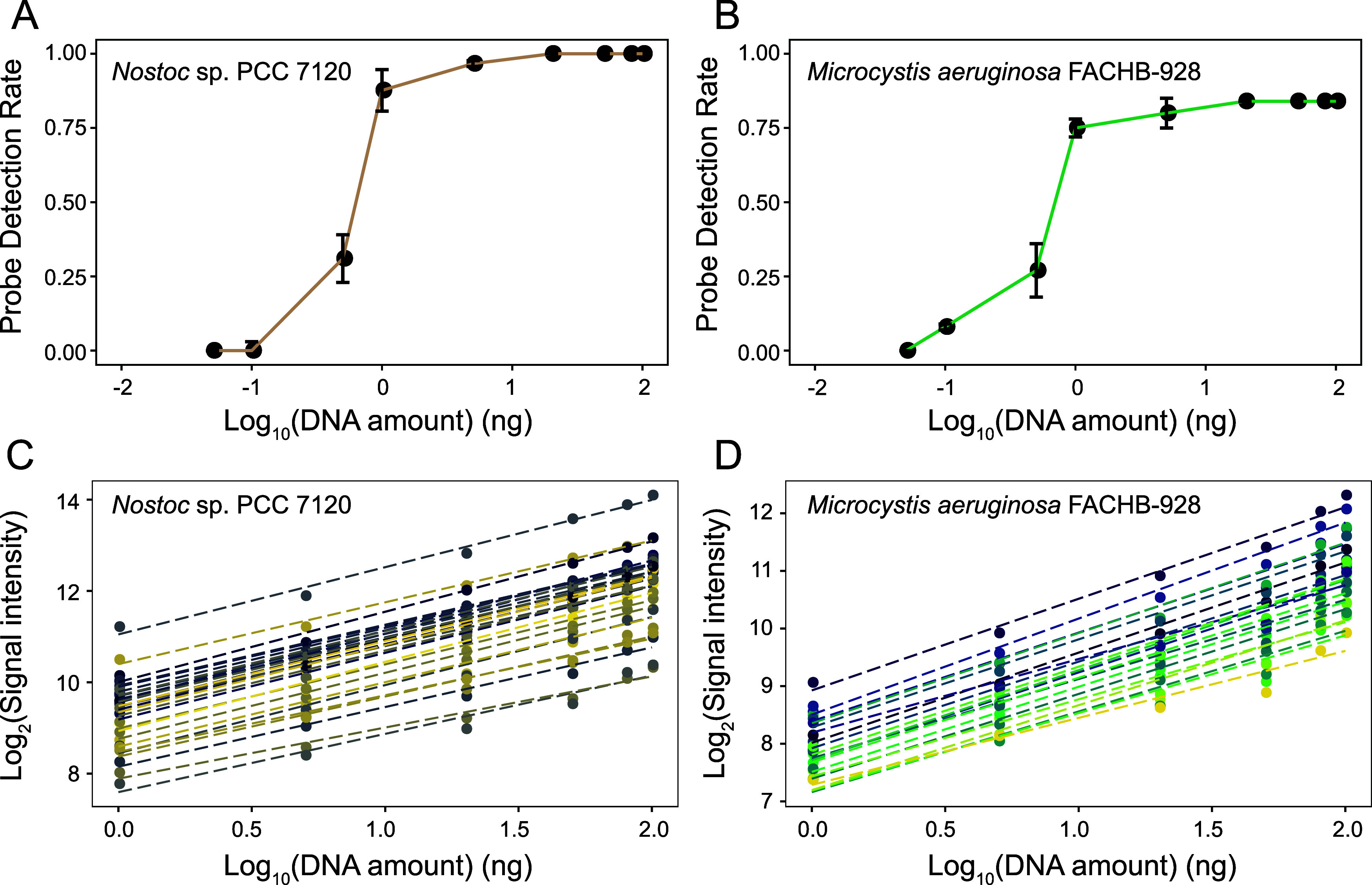
Sensitivity and quantitative performance evaluation. Relationships between PDR values and DNA amounts (0.05 to 100 ng) for *Nostoc* sp. PCC 7120 (A) and *Microcystis aeruginosa* FACHB-928 (B). Linear correlations between the signal intensities and DNA amounts for all probes of the *Nostoc* sp. PCC 7120 (C) and *Microcystis aeruginosa* FACHB-928 (D).

To determine the detection limit of CyanoStrainChip, we conducted *in vitro* tests using genomic DNAs from *Microcystis aeruginosa* FACHB-928 and *Nostoc* sp. PCC 7120. At a genomic DNA concentration of 1 ng, we observed that over 84% of probes targeting *Nostoc* sp. PCC 7120 and over 75% of probes targeting *Microcystis aeruginosa* FACHB-928 had significant fluorescence intensities, implying true positive detections based on the threshold of PDR 0.75 (see Figure 4A, B). When the DNA concentration was equal to or greater than 5 ng, the PDR values of both strains entered the platform period. Meanwhile, a small percentage of probes (5% of *Nostoc* sp. PCC 7120 and 8% *Microcystis aeruginosa* FACHB-928) were detected even at a genomic DNA concentration of 0.5 ng, representing a certain degree of positive hybridization.

Although 50-base-long probes are believed to achieve high hybridization sensitivity and efficiency,^46^ the sensitivity limit of CyanoStrainChip is relatively high compared to other molecular tools for microorganism detection. For instance, an oligonucleotide microarray in order to detect 17 different wine-spoilage microorganisms was able to detect target DNA obtained from LATE-PCR performed with 0.4 pg of the template.^47^ We figure that this is because conventional molecular methods for microorganism detection primarily rely on PCR amplification of gene markers, whereas the library preparation of CyanoStrainChip is PCR-free. In recent years, whole genome amplification techniques have facilitated the metagenomics study of low biomass environments.^48^ This has led to the belief that the introduction of such an amplification step could help CyanoStrainChip lower its detection limit. However, it should be noted that whole genome amplification techniques indeed have some disadvantages, such as nonuniform amplification across the genome, poor repeatability, and external contamination.^49^ Consequently, there is a trade-off between sensitivity and the risk of false-positive detection.

### 3.4 Semi-Quantitative Evaluation

The quantitative performance of CyanoStrainChip was evaluated by measuring the fluorescence intensities of serially diluted genomic DNAs (1, 5, 20, 50, 80, and 100 ng) from pure cultures of *Microcystis aeruginosa* FACHB-928 and *Nostoc* sp. PCC 7120. The resulting fluorescence intensities of all specific probes targeting the spiked strains displayed a linear growth rate proportional to the increase in the DNA amount (see Figure 4C, D). This was further supported by linear regression analysis (36 probes for *Microcystis aeruginosa* FACHB-928, *R* = 0.978 to 0.999; 30 probes for *Nostoc* sp. PCC 7120, *R* = 0.995 to 0.999, see Table S4). These outcomes recommend that our CyanoStrainChip has the potential to be a highly quantitative tool for comparing the abundance of the same strain across multiple samples. Additionally, we observed a significant difference in the average fluorescence intensities between the two strains at the same DNA amount. Therefore, we proposed that CyanoStrainChip is not directly quantitative, but rather semiquantitative, and quantitative abundances can primarily be assessed between samples or over time of the same taxa, but not strictly between different taxa in one sample.

Accurate quantitation of cyanobacteria from complex environmental samples is of vital importance; Nevertheless, current molecular detection tools have insufficient quantitative capacity. The sequencing of the 16S rRNA gene generated by PCR amplification to detect cyanobacteria has been the most popular methodology for the past ten years. The effects of PCR amplification and different 16S gene copy numbers in different microbiomes can lead to bias in the quantitative measure of cyanobacteria dynamics.^50,51^ As for metagenomics, strain level quantification is quite difficult because the majority of reads from closely related strains will go toward identical genomic regions, and their nonconserved regions are not covered at a sufficiently high depth to quantify the relative abundances.^21^ Similar to the quantitative abilities of previous other DNA arrays,^23−28^ CyanoStrainChip showed a high linear correlation between strain fluorescence intensities and concentrations using *in vitro* tests and thus can make up the shortcomings in quantitative measure effectively of existing cyanobacterial detection techniques. At the same time, the application of CyanoStrainChip may be somewhat limited due to the semiquantitative nature of DNA microarrays, which have been widely discussed in the past 20 years.^52−54^

### 3.5 Field Study of Harmful Cyanobacterial Blooms in Lake Chaohu

**Figure 5:**
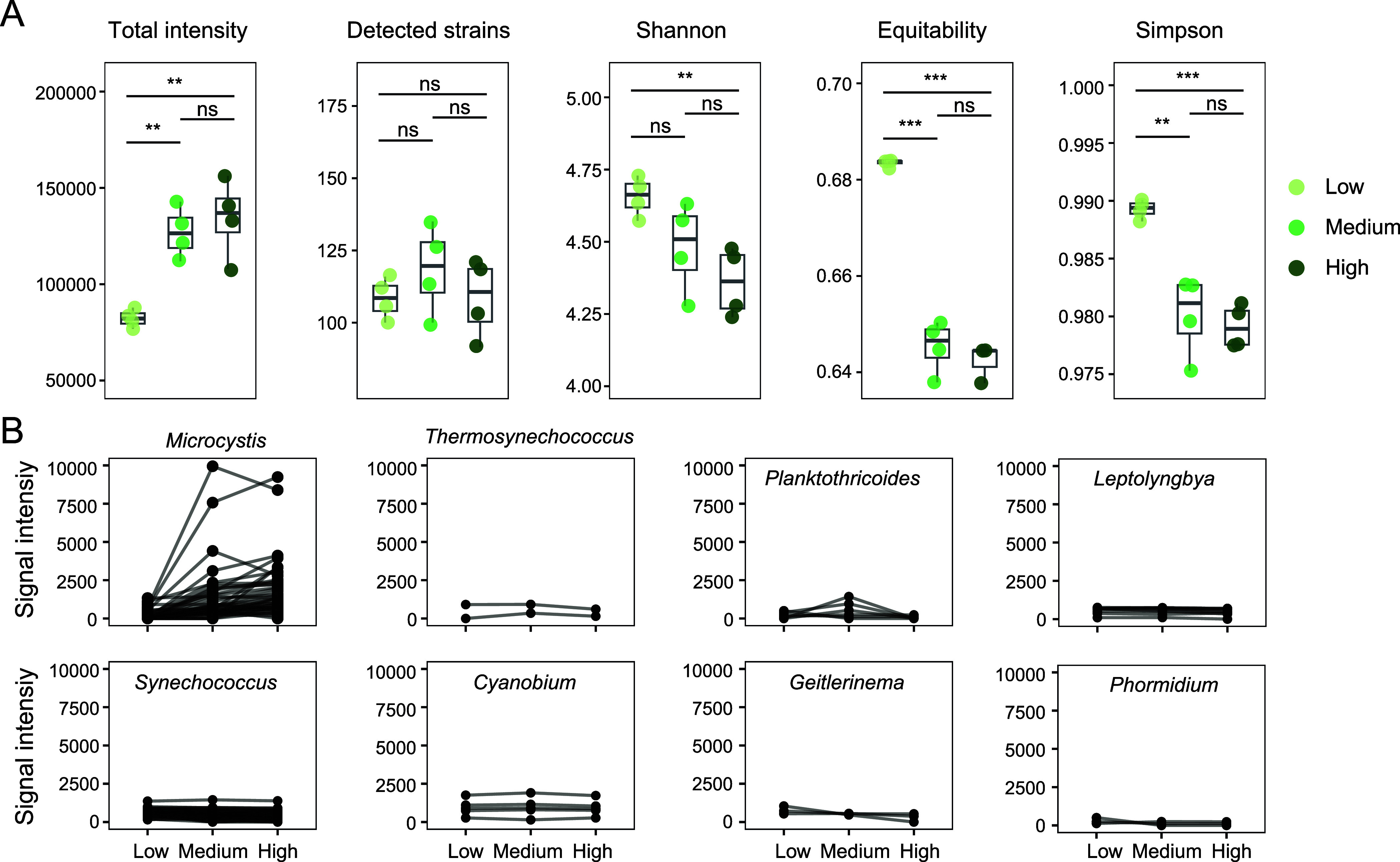
CyanoStrainChip analysis of water samples from different severity levels of cyanobacterial blooms in Lake Chaohu. (A) Comparison of alpha diversity metrics between samples from sites with different severity levels of blooms. The water samples were labeled as “Low,” “Medium,” or “High” based on their chlorophyll concentration of 17.7, 74.1, and 144.2 μg/L, respectively. Comparisons were performed using a Mann–Whitney test (*P < 0.05, **P < 0.01, ***P < 0.001). (B) Alteration of cyanobacterial strains belong to dominant genera between different severity levels of blooms.

To test the practicality of CyanoStrainChip for detecting cyanobacteria in complex environments, we conducted a field study of harmful cyanobacterial blooms in Lake Chaohu. Twelve water samples were collected from the surface water of harmful cyanobacterial blooms at three sites in Lake Chaohu with third-level chlorophyll concentrations for representing the severity levels of blooms. The water samples were labeled as “Low,” “Medium,” or “High” based on their chlorophyll concentration of 17.7, 74.1, and 144.2 μg/L, respectively. A total of 161 cyanobacterial strains were identified in all samples using a detection threshold of PDR 0.75 (details of CyanoStrainChip detections in Table S5). We found that as the chlorophyll concentration increased, the sum total of the normalized intensity exhibited a growing trend. However, the number of identified strains in different groups did not change conspicuously (Figure 5A). Most notably, we found that the alpha diversity of the cyanobacterial communities significantly decreased as the severity levels of cyanobacterial blooms increased (Figure 5A). It is in accordance with the results of previous studies, which demonstrated that alpha diversity of the cyanobacterial community was significantly decreased during cyanobacterial bloom outbreaks.^55,56^ The explanation for this phenomenon is that the bloom is mainly attributed to the overproduction of a narrower range of cyanobacterial taxa. To prove this, we analyzed the alternation of cyanobacterial strains belonging to the dominating genera, which is shown in Figure 5B. The genus *Microcystis* displayed the most notable trends, with the majority of its strains exhibiting a clear increase in abundance as chlorophyll concentrations rose. Conversely, none of the other genera displayed a pattern comparable to that of *Microcystis*. Comparison of these findings with those of other studies confirms that *Microcystis* is the predominant bloom-forming genus in Lake Chaohu.^57,58^

Another remarkable observation in the CyanoStrainChip results was the variability of abundance change among strains within the same genus between different severity levels of blooms. Despite the overall trend of *Microcystis* being an increase, certain strains displayed a steady tendency. For example, *Microcystis* sp. FACHB-1515 and *Microcystis aeruginosa* FACHB-1023 were detected in all samples with high PDR values, and their intensities stabilized around 1000. Furthermore, some *Microcystis* strains showed an apparent stronger uptrend compared to others such as *Microcystis aeruginosa* KW and *Microcystis aeruginosa* NIES-843. These two strains showed intensities around 500 in low samples, while this value drastically increased to around 9000 in Medium and High samples. We thus speculate that these two strains have a fairly high probability of being the most important cyanobacteria related to the outbreaks of Lake Chaohu harmful blooms. These results are consistent with previous research which found that the season succession of the *Microcystis* population in Lake Taihu was dominated by a few genotypes despite there being over 20 genotypes have been identified.^59^ Therefore, we propose that future efforts to suppress harmful cyanobacterial blooms in Lake Chaohu should focus on specific strains rather than at the species or above-species level.

### 3.6 Comparison of CyanoStrainChip to Next-Generation Sequencing

The most widespread technique for identifying what cyanobacterial species are present in an environmental sample is next-generation sequencing, including 16S rRNA and short-gun metagenomic sequencing. Herein, we compared the outcomes of CyanoStrainChip analysis and 16S rRNA sequencing on twelve field samples from harmful cyanobacterial blooms in Lake Chaohu. The results suggested robust correlations between alpha diversity indices obtained through CyanoStrainChip and 16S rRNA sequencing (refer to Figure S6A–C. Total cyanobacterial abundance, *R* = 0.767, *P* < 0.001; Shannon index, *R* = 0.591, *P* < 0.001; Simpson index, *R* = 0.923, *P* < 0.001). In terms of cyanobacterial composition, the two methods identified 10 common families, while eight families and one family were uniquely identified by 16S rRNA sequencing and CyanoStrainChip, respectively (Figure S6D). It is noteworthy that these families identified uniquely by each method contribute minimally to the overall cyanobacterial community abundance (Figure S6E), implying that the more abundant and ecologically significant cyanobacteria were successfully detected by both methods. Furthermore, correlation analysis of the abundance of major cyanobacterial families across all samples in the results of both methods revealed a high degree of similarity (Figure S6F, *R* = 0.844, *P* < 0.001). These results indicate the concordance in cyanobacterial identification between CyanoStrainChip and 16S rRNA sequencing, particularly for predominant cyanobacterial taxa. However, it is crucial to highlight the disparities in analytical outcomes between the two methods, that is 16S rRNA sequencing struggles to achieve strain-level resolution compared to CyanoStrainChip. There were 172 cyanobacterial OTUs identified by 16S rRNA sequencing, with only 39 OTUs assigned to the genus level. Among them, merely four OTUs belonged to the genus *Microcystis*, whereas CyanoStrainChip detected dozens of distinct *Microcystis* strains. While both methods underscored the significance of the genus *Microcystis* in the Chaohu cyanobacterial bloom outbreaks, only CyanoStrainChip could pinpoint the specific strains. It truly matters that definite functional traits can be assigned to a specific strain rather than a genus. For instance, while the toxicity of a cyanobacteria strain generally remains consistent, the toxicities of *Microcystis* vary. We successfully used antiSMASH to evaluate the potential release of eight common cyanotoxins of all target strains, demonstrating the possibility to connect the CyanoStrainChip outcomes to the specific trait by utilizing available genomic resources (see Supporting Information).^60^ This advantage enables CyanoStrainChip to facilitate trait-based studies, potentially uncovering novel insights that are unattainable with an OTU-based approach.^61^

**Figure 6:**
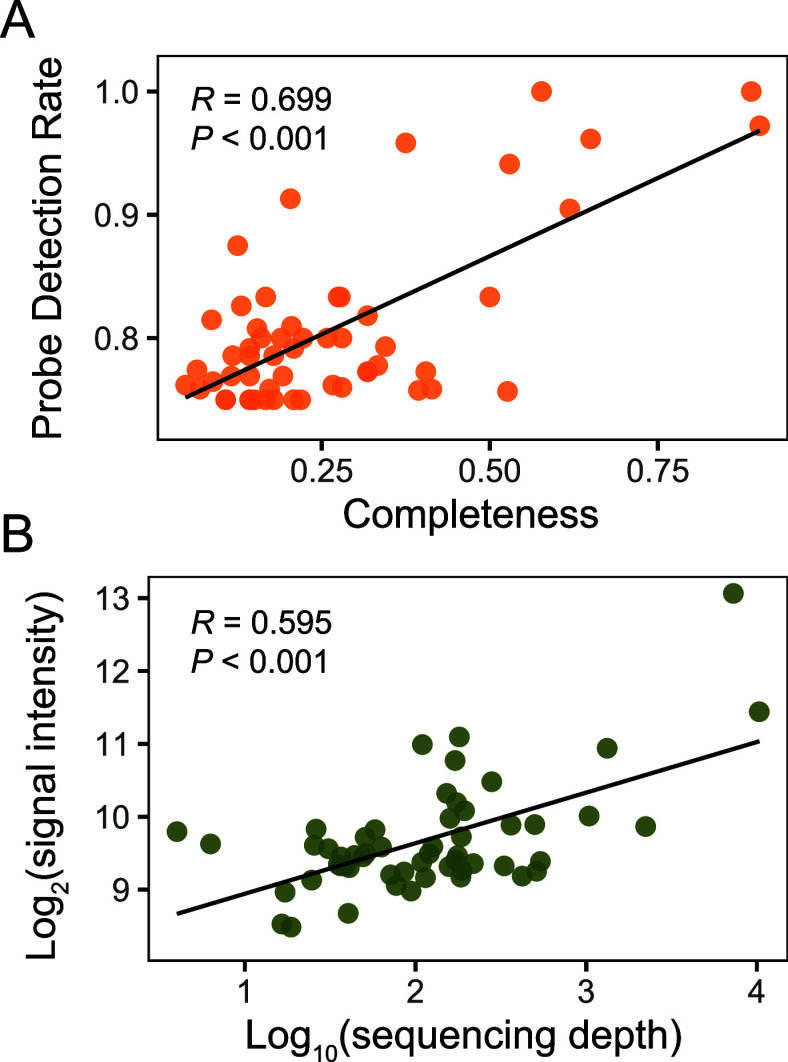
Comparison of CyanoStrainChip to shotgun metagenomic sequencing using a water sample of cyanobacterial bloom from Reservoir Dashahe. (A) Scatter plots of detected strains in completeness analyzed by metagenomic sequencing (*x*-axis) and Probe Detection Rate (PDR) analyzed by CyanoStrainChip (*y*-axis). (B) Scatter plots of detected strains in sequencing depth analyzed by metagenomic sequencing (*x*-axis) and fluorescence intensities analyzed by CyanoStrainChip (*y*-axis).

Subsequently, a comparative examination of the cyanobacterial profiles of a water sample from the Reservoir Dashahe cyanobacterial bloom obtained through CyanoStrainChip and metagenomic short-gun sequencing was conducted. In advance, it is essential to clarify that in the metagenomic analysis, the completeness of each strain was counted by directly mapping our strain-specific probes to the metagenome assembly results, and the average sequence depth of the mapped probe was used to represent the abundance of detect strains. As Figure 6 shows, there were significant positive correlations between CyanoStrainChip and metagenomic results in terms of both the completeness and abundance of detected strains. These findings suggest that the cyanobacterial community profiles obtained by the two methods are generally consistent with each other. However, some differences between the results of the two methods were observed and should be mentioned. A Venn diagram was used to display the overlap of the top 20 abundant detected cyanobacterial strains between CyanoStrainChip and metagenomic sequencing (see Figure S7). Only half of these strains were among the top 20 members identified by both methods. For example, *Aphanizomenon flos-aquae* FACHB-1416 can be detected by CyanoStrainChip with a fairly high PDR of 0.913, while only 20% of its specific probes can be mapped to metagenome assembly. Conversely, 63% of *Cylindrospermopsis raciborskii* CENA303 specific probes exist in the metagenome assembly, while the PDR yield by CyanoStrainChip is 0.34, considerably lower than the detection threshold. These findings support the idea proposed in previous studies that combining the two approaches could achieve synergies.^20,29^

### 3.7 Implications and Considerations

Collectively, the novel DNA microarray CyanoStrainChip presented in this study enables accurate and high-throughput detection of environmental cyanobacteria at the strain level. The application of CyanoStrainChip can provide novel insights into the ecological study and harmful bloom management attributed to its unique advantages (see Table S7, a summary of the advantages and disadvantages of existing methods). Compared to the traditional isolation and culture method, CyanoStrainChip is culture-independent and only requires DNA of the environmental sample, which means it can be less time-consuming. The use of PCR-based amplicon sequencing techniques has revolutionized cyanobacterial biology in the last decades; however, it often causes ambiguity in taxonomic classification due to the limitation of the sequencing length. Compared to metagenomic sequencing of environmental samples for cyanobacterial detection, CyanoStrainChip analysis has lower cost, lower computational requirement, and better quantitative effect. Also, this new tool exhibits a higher detection throughput in comparison to immunological methods.

Meanwhile, as a “closed format” tool, CyanoStrainChip is capable of detecting a predetermined set of targets (1277 cyanobacterial strains in the current version) and is not suitable for novel cyanobacteria explorations. This is mainly due to the pervasive problem of oligonucleotide microarrays: cross-hybridization. For unknown cyanobacterial strains, the calculation of their PDR values for false-positive control is computationally infeasible, because their genome sequences are unavailable. While this limitation hinders a comprehensive exploration of cyanobacteria, it concurrently mitigates the risk of spurious errors. On one hand, given the rapid accumulation of cyanobacterial genomic data, it is imperative to continually update CyanoStrainChip to incorporate a broader spectrum of cyanobacteria in the future. On the other hand, it is advisable to integrate CyanoStrainChip with high-throughput sequencing methods (e.g., metagenomics sequencing, 16S rRNA sequencing), which may facilitate a more comprehensive approach to understanding cyanobacterial biology and ecology. Moreover, this work serves as a good model for how to leverage the power of microarray and genomic knowledge accumulation to customize detection tools for a specific group of microorganisms at high taxonomic resolution.

## Supporting Information Available

Strain-specific probe design; preprocessing of CyanoStrainChip data; 16S rRNA sequencing analysis; annotation of genome and prediction of toxin biosynthesis gene clusters; ROC and PR analysis; validation of using the spike-in control for CyanoStrainChip data normalization; diagram of the preprocessing steps for CyanoStrainChip data; computational evaluation of the remained probes; location of probes on CyanoStrainChip in genomes; utility of the spike-in control for CyanoStrainChip data normalization; evaluation of the effectiveness of CyanoStrainChip under the addition of background DNA; comparison of CyanoStrainChip to 16S rRNA sequencing; venn diagram of the top 20 abundant cyanobacterial strains uncovered by CyanoStrainChip and metagenomic sequencing; statistical results of antiSMASH predictions of the toxicities of target cyanobacteria strains; summary of used strains in mock communities; summary of strain-specific probes in CyanoStrainChip organized by main taxonomic groups; *R* values for linear correlation of the signals of all specific probes to the total amounts; the detected cyanobacterial profiles of blooms at three sites in Lake Chaohu; detected cyanobacterial profiles of one water sample from Reservoir Dashahe; and summary of advantages and disadvantages of methods for detection of environmental cyanobacteria (PDF)
Detailed information on the 1277 used strains on the CyanoStrainChip (XLSX)
Predictions of toxicities of the 1277 used strains on the CyanoStrainChip (XLSX)
