# Supplementary material for: CyanoStrainChip: A Novel DNA Microarray Tool for High-Throughput Detection of Environmental Cyanobacteria at the Strain Level
Source: Environ Sci Technol. 2024 Mar 8;58(11):5024–34. doi: 10.1021/acs.est.3c11096 (PMC10956431; doi:10.1021/acs.est.3c11096)
Supplement: Supplementary file 1 — es3c11096_si_001.pdf [file es3c11096_si_001.pdf]

Supporting Information for:

CyanoStrainChip: A novel DNA microarray tool for high-throughput detection of environmental cyanobacteria at the strain level

Hao-Yue Shu[1,2], Liang Zhao[3], Yanyan Jia[4], Fei-Fei Liu[1], Jiang Chen[1], Chih-Min Chang[1], Tao Jin[1,5,], Jian Yang[2,*], Wen-Sheng Shu[1,3,*]*

[1]Guangdong Magigene Biotechnology Co., Ltd., Shenzhen 518081, PR China

[2]School of Food and Drug, Shenzhen Polytechnic, Shenzhen 518081, PR China

[3]Institute of Ecological Science, Guangzhou Key Laboratory of Subtropical Biodiversity and Biomonitoring, Guangdong Provincial Key Laboratory of Biotechnology for Plant Development, School of Life Sciences, South China Normal University, Guangzhou 510006, PR China

[4]School of Ecology, Sun Yat-sen University, Shenzhen 518081, PR China

[5]One health Biotechnology (Suzhou) Co., Ltd., Suzhou, 215009, PR China

[*]Corresponding authors: Wen-Sheng Shu, shuwensheng@m.scnu.edu.cn; Jian Yang, jiany@szpt.edu.cn; Tao Jin, jintao@magigene.com

This SI contains 6 supporting texts, 8 figures and 6 tables, in total 35 pages.

Table of Contents

Texts:

Supplementary Method S1. Strain-Specific Probe Design

Supplementary Method S2. Preprocessing of CyanoStrainChip Data

Supplementary Method S3. 16S rRNA Sequencing Analysis

Supplementary Method S4. Genome Annotation and Prediction of Toxin Biosynthesis Gene Clusters

Supplementary Method S5. ROC and PR analysis

Supplementary Results and Discussion S1. Utility of The Spike-In Control for CyanoStrainChip Data Normalization

Figures:

Figure S1. Diagram summarizing the preprocessing steps for CyanoStrainChip data

Figure S2. Computational evaluation of the remained probes

Figure S3. Location of probes on CyanoStrainChip in genomes

Figure S4. Utility of the spike-in control for CyanoStrainChip data normalization

Figure S5. Evaluation of the effectiveness of CyanoStrainChip under the addition of a complex background DNA

Figure S6. Comparison of CyanoStrainChip to 16S rRNA sequencing using water samples of cyanobacterial blooms from Chaohu

Figure S7. Venn diagram of the top 20 abundant cyanobacterial strains uncovered by CyanoStrainChip and metagenomic sequencing

Figure S8. Statistical results of antiSMASH predictions of the toxin-producing features of all 1,277 cyanobacteria strains

Tables:

Table S2. Summary of used strains in mock communities

Table S3. Summary of strain-specific probes in CyanoStrainChip organized by main taxonomic groups

Table S4. R values for linear correlation of the signals of all specific probes to the total amounts of target DNA for Microcystis aeruginosa FACHB-928 and Nostoc sp. PCC7120

Table S5. The detected cyanobacteria profiles of cyanobacteria blooms at three sites in Lake Chaohu with third-level chlorophyll concentrations

Table S6. The detected cyanobacteria profiles of one water sample from Reservoir Dashahe by the CyanoStrainChip and metagenomic short sequencing

Table S7. Summary of advantages and disadvantages of methods for detection of environmental cyanobacteria

Supplementary Method S1. Strain-Specific Probe Design. In principle, each probe was designed to target a unique region of only one specific strain, and each strain was expected to contain dozens of probes to improve the accuracy and sensitivity of detection. We performed a *k*-mer based method to discover strain-specific oligonucleotides, as described by Tu et al, 2015. with minor modifications. Initially, we enumerated 50mers from all cyanobacterial genomes and used jellyfish (Marçais and Kingsford, 2011) and bowtie2 (Langmead and Salzberg, 2012) to count their frequency and track their source. We extracted *50*-mers that occurred in only one

strain with a frequency less than twice. As contiguous matching stretches are critical to the cross-hybridization of probes with non-target sequences, each reserved *50*-mer was divided into *20*-mers, and we used bowtie2 to align all *20*-mers to all cyanobacterial genomes. We discarded the *50*-mer that contained a *20*-mer with valid alignment in more than one genome. Non-target sequences that have >75−80% sequence similarity with probes may cause cross-hybridization, as shown by Kane et al, 2000. We ran Megablast to search for the closest sequences of remaining *50*-mers. If a *50*-mers had blast hits over 80% total identity from more than one genome, it was discarded. The remaining *50*-mers were considered as candidate probes. We further refined candidate probes based on various oligonucleotide properties using criteria described previously (Li et al., 2005; Li et al., 2015; Shi et al., 2019). For complexity, DustMasker was utilized to identify and eliminate low-complexity probes. For probe secondary structure screening, the probes with self-folding energy lower than –4 kcal/mol, dimer score higher than 5, and hairpin score higher than 6 were discarded. The remaining probes underwent a specificity check by searching against the NCBI GenBank NT database, excluding cyanobacterial nucleotide sequences, with the criteria of a blast score < 34. In terms of binding free energy, melting temperature, and GC content, we initially set defined thresholds for the preliminary screening (melting temperature: 65 to 82 °C; GC content: 0.3 to 0.75; Binding free energy to its target: -85 to -60 kcal/mol). Subsequently, we employed piece-wise linear functions in iterative processes to select probes within the minimal interval of oligonucleotide properties for each strain while maintaining an adequate number of probes (Li et al., 2005).

Supplementary Method S2. Preprocessing of CyanoStrainChip Data. We took several microarray data preprocessing steps to offset systematic variation and filter false positive detections before downstream analysis (see Figure S1). The raw fluorescent signal of each probe

were firstly background corrected by subtract the background signal from the foreground signal. We chose g(r)MeanSignal which was the mean signal of spot from inlier pixels in green and/or red channel as foreground signal, and gBGMeanSignal which was the mean local background signal (local to corresponding spot) computed per channel (inlier pixels) as background signal. Subsequently, we used spike-in control as standards to fit a loess model to normalize background corrected data by loess.normalize function in R package affy (Gautier et al., 2004). For this purpose, the matrix containing the background corrected probe signal intensities was set to mat parameter, while the matrix containing the background corrected spick-in control probe signal intensities was set to subset parameter. The rest parameters were set to the default values. After normalization we identified and removed undetected probes based on AFE generated features including IsWellAboveBG and IsPosAndSignif. The Boolean flag of IsPosAndSignif was determined by the different significance tests between foreground and the background mean signal. If P-value < 0.01, and foreground MeanSignal > background mean signal, then such a probe was marked as 1 under this feature. IsWellAboveBG was used to indicate whether the probe background-subtracted signal is well above the background. If the background-subtracted signal is greater than the 5 times background standard deviation, then the probe gets a Boolean flag of 1 under this feature. Probes passed the two criteria were regarded as detected probes. However, these detected probes may exceed the number of "true positive" probes because of the potential cross-hybridization reaction of probes with non-target DNA sequence. To reduce the false positives, we have defined a simple metric for strain detection: Probe Detection Rate (PDR), which can be calculated with this equation: PDR = the number of detected probes of one strain/ all designed specific probes of one strain. Our presumption is that dozens of probes from one

strain have significant signals due to cross-hybridization event at the same time has a very small probability.

Supplementary Method S3. 16S rRNA Sequencing Analysis. The paired-end reads were merged using Mothur (v.1.2.11) (Schloss et al., 2009), and subsequent processing and analysis of raw reads were performed using QIIEM (v.1.9.1) (Caporaso et al., 2010) to eliminate low-quality reads. Following quality control, sequences were clustered into operational taxonomic units (OTUs) via UPARSE (v.7.0.1090) (Edgar, 2013), employing a 97% sequence similarity threshold. A representative sequence from each OTU was matched against the SILVA databases (Release 138). To make the 16S rRNA sequencing results comparable to CyanoStrainChip, taxonomic classifications underwent a thorough double-check against the representative genomes of 1,277 strains. Unclassified Operational Taxonomic Units (OTUs) were excluded, and the filtered sequence data were normalized to alleviate sequencing biases, enabling a comprehensive comparison of community variations.

Supplementary Method S4. Genome Annotation and Identification of Toxin Biosynthesis Gene Clusters. The Open reading frames (ORFs) of 1,277 cyanobacterial genomes were predicted using Prodigal (Hyatt et al., 2010) and the resulting protein sequences from respective genome were compared to Database of Clusters of Orthologous Genes (COGs) by using DIAMOND (Buchfink et al., 2015) for functional annotation. Subsequently, for each strain, their probe loci were compared with the loci of protein-coding genes, followed by the categorization of all probes into three types: those completely within the gene region, those partially within the gene region, and those outside the gene region. For probes which located entirely within the gene region, we tabulated the counts of relevant genes in the COG categories and assessed the enrichment levels of these genes in each category relative to the overall gene set. Furthermore,

we used command-line version of antiSMASH 5 (Blin et al., 2019) with the bacterial setting and otherwise default parameters to predict the secondary metabolite biosynthesis gene clusters (BGCs) of all genomes. Subsequently, these BGCs were compared to the Minimum Information about a Biosynthetic Gene cluster (MIBiG) database (Kautsar et al., 2020) to annotate the compounds that would be produced by each region. The strains containing BGCs of eigh common cyanobacterial toxins (anatoxin-a, curacin, cylindrospermopsin, hectochlorin, jamaicamide, microcystin, nodularin, and saxitoxin) were recorded, and probes located within these regions were counted and documented. However, be aware that the detection of cyanotoxin BGCs heavily relies on existing databases of known BGCs. This implies that strains lacking toxin BGC annotations in this context still hold the potential to produce novel toxins beyond the established set of eight.

Supplementary Method S5. ROC and PR analysis. To evaluate the effectiveness of strain detection using the CyanoStrainChip, Precision-Recall (PR) and Receiver Operating Characteristic (ROC) curves were computed on the mock community experiments data by varying the PDR threshold. The resulting matrix of samples and strains was compared to the DNA composition of the mock communities. This allowed for the counting of true positives, false positives, true negatives, and false negatives. A true positive (TP) was defined as the detection of a strain in a sample when the strain's DNA was actually spiked in the sample. A false positive (FP) occurred when a strain was detected in a sample, but the strain's DNA was not actually spiked in the sample. True negatives (TN) were strains that did not exist in the samples and did not pass the PDR threshold. False negatives (FN) were strains that existed in the samples but did not pass the PDR threshold. A ROC curve is the plot of the true positive rate (TPR = TP/(TP+FN)) against the false positive rate (FPR = FP/(FP+TN)) at each threshold setting. A PR

curve is simply a graph with Precision values on the y-axis and Recall values on the x-axis. In other words, the PR curve contains TP/(TP+FP) on the y-axis and TP/(TP+FN) on the x-axis. Herein, ROC and PR curves as well as the areas under ROC curve and PR curve were calculated using the PRROC (v1.3.1) package in R (Grau et al., 2015).

Supplementary Results and Discussion S1. Utility of The Spike-In Control for CyanoStrainChip Data Normalization. As proposed by authors Chen et al., 2015, exogenous spike-in controls should be needed in all types of genome-wide profiling experiments when the distribution of targets between compared samples is asymmetric. In this study, we implemented a set of probes targeting synthetic DNAs to serve as spike-in control for microarray experiments. To verify the validity of the spike-in control, we prepared mock cyanobacterial communities samples by combining the spike-in synthetic with strain *Microcystis aeruginosa* PCC 7806SL (30 ng), *Microcystis aeruginosa* FACHB-928 (range: 1 to 100 ng), and *Nostoc* sp. PCC 7120 (range: 1 to 100 ng). For *Microcystis aeruginosa* FACHB-928 and *Nostoc* sp. PCC 7120, their fluorescence intensity as a function of DNA amount were plotted in Figure S4A and B. From the chart, it can be seen that spike-in control normalized approaches improved Pearson's correlation coefficients for both strains compare to raw data; meanwhile the global normalized approaches significantly changed the slope, but did not improve Pearson's correlation coefficients. For strain *Microcystis aeruginosa* PCC 7806SL, the amount of added DNA was consistent in all samples, and we observed a fluctuation of non-normalized signals across all samples that implied systematic experimental bias and technical variation. Figure S4C compares the spike-in control normalized and global normalized signals to the raw signals of *Microcystis aeruginosa* PCC 7806SL, highlighting the dramatic decline of variability in spike-in control normalized data. In contrast, global normalized approaches appeared to introduce new errors. Moreover, to

determine the applicability of the spike-in control for CyanoStrainChip analysis of complex environmental samples, we analyzed three water samples from Lake Chaohu and one water sample from Reservoir Dashahe, each with four technical replicates. The signals of all positive probes for each sample were normalized by spike-in control probe signals. The box plots of variations of all probes within replicates before and after normalization were compared and shown in Figure S4D. It is shown that the variations of replicates for each sample were significantly lower after normalization by the spike-in control, indicating a successful reduction of system bias.

A typical microarray experiment result can have numerous sources of variation, which may be attributed to biological and non-biological causes. To accurately detect underlying biological variation, normalization of oligonucleotide microarray data has become a standard procedure that offsets non-biological differences between samples. There are several methods for normalizing microarray data, with global normalization being the most commonly used (Zahurak et al., 2007). However, the fundamental assumption for global normalization is that different samples have the same abundance of total targets, which is not applicable to actual field data (Chen et al., 2015). *In vitro* mock community experiments have demonstrated that global normalization approaches cannot remove non-biological effects or may even introduce new errors when the total added DNA amount of targets differs (Figure S4A, B, and C). Therefore, to overcome this limitation, spike-in controls are needed. Spike-in control has been shown to be valid in the detection of environmental microbes using microarray (Liang et al., 2010), 16S rRNA sequencing (Tourlousse et al., 2017), and metagenomics sequencing (Kalantar et al., 2020). In this study, we designed a set of control probes for targeting synthetic DNAs that were uniformly distributed in the CyanoStrainChip array. Analysis of mock community samples and field samples has shown

that this approach is reliable and reproducible, enabling accurate normalization of CyanoStrainChip data.

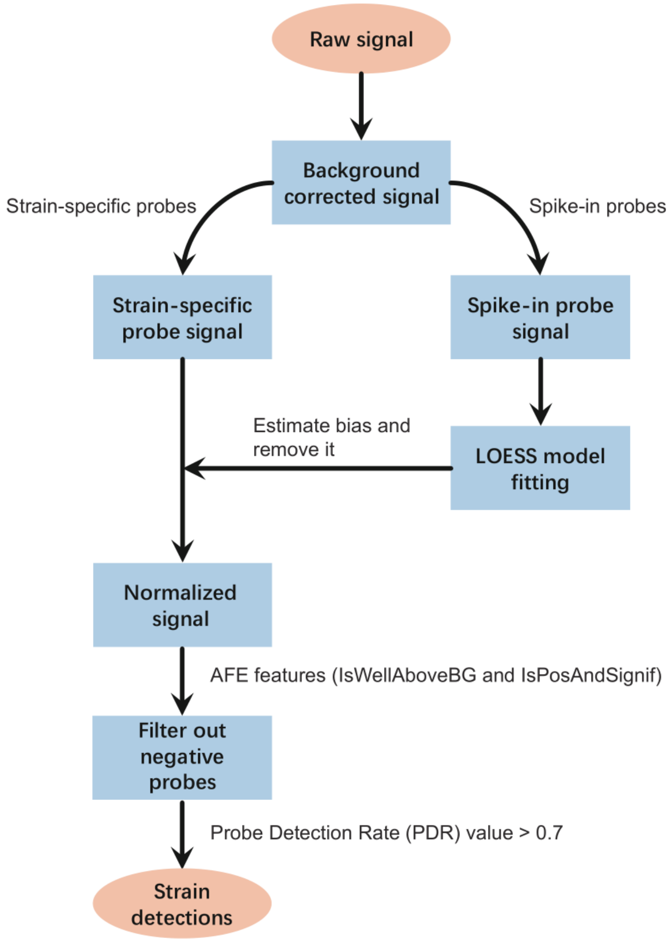

Figure S1. Diagram summarizing the preprocessing steps for CyanoStrainChip data.

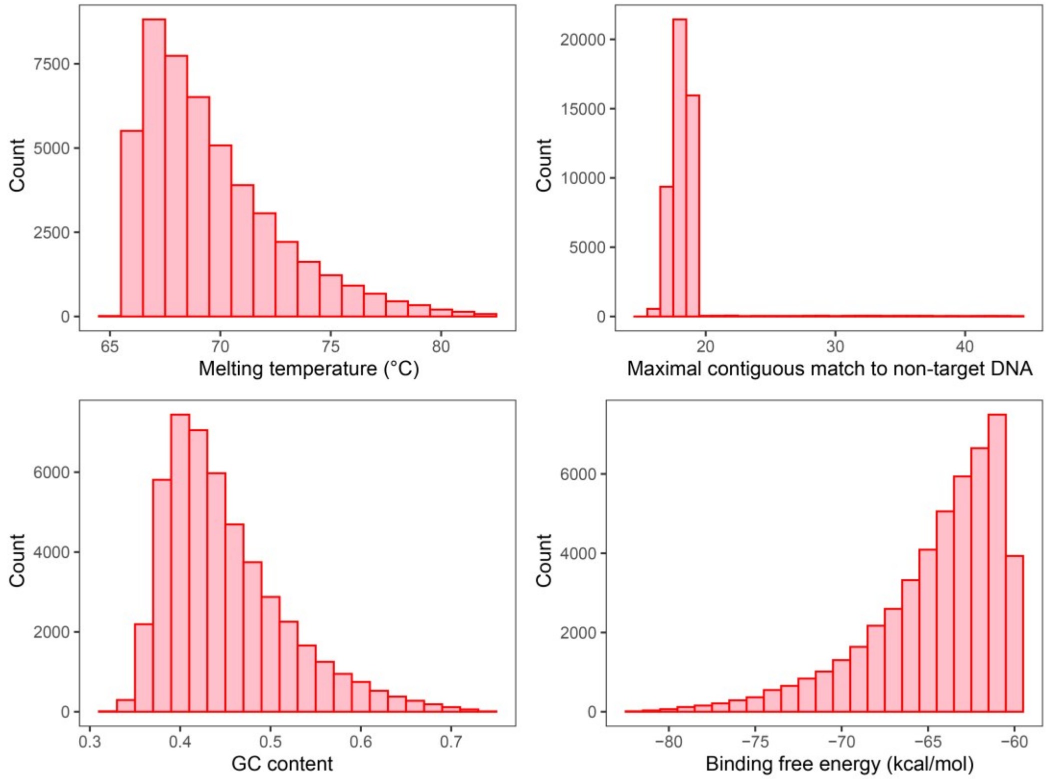

Figure S2. Computational evaluation of the specificity of the remained probes based on binding free energy, GC content, melting temperature, and contiguous similarity to non-target genomes.

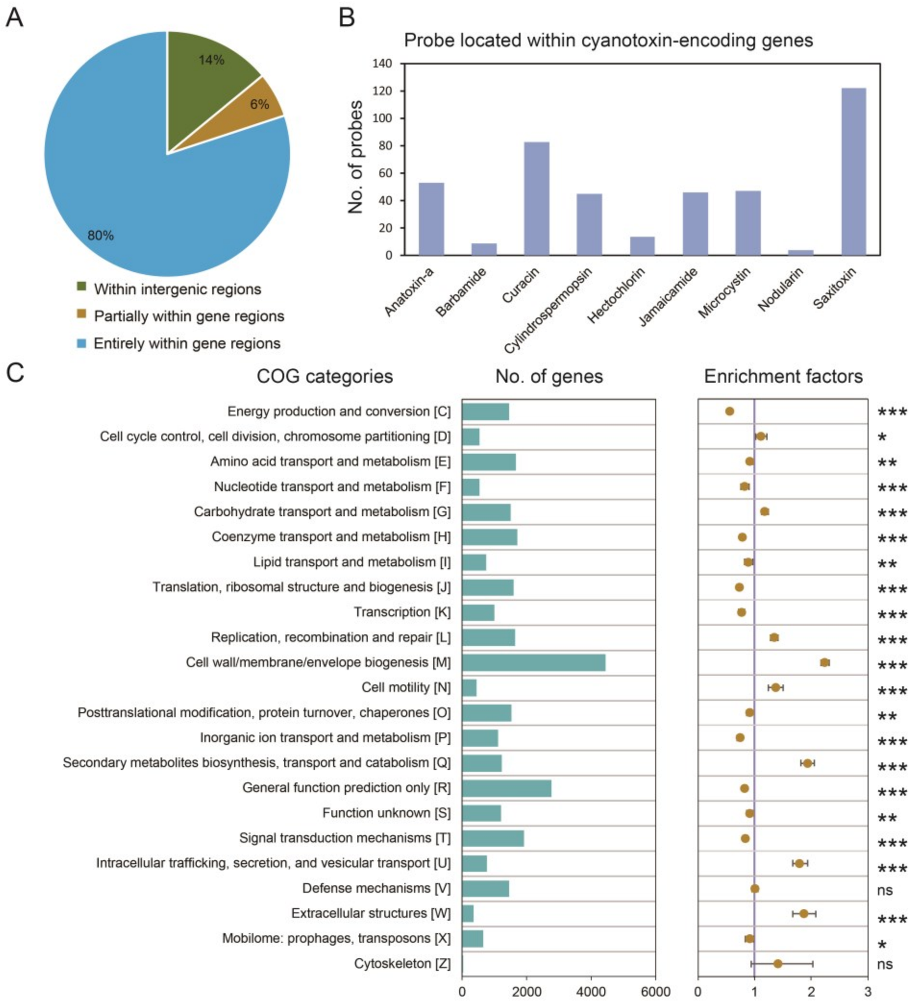

Figure S3. Location of probes on CyanoStrainChip in genomes. (A) Proportional distribution of the probes located within gene regions and intergenic regions. (B) Distribution of the probes located within cyanotoxin-encoding genes. (C) Distribution and enrichment analysis of genes where the probes are located based on COG functional categories. The enrichment factors of probe associated genes within a COG category was calculated by comparison to overall gene set. The significant differences were calculated using the two-tailed Fisher's exact test and indicated by asterisks as follows: ns, $P \geqslant 0.05$; *, $P < 0.05$; **, $P < 0.01$; ***, $P < 0.001$.

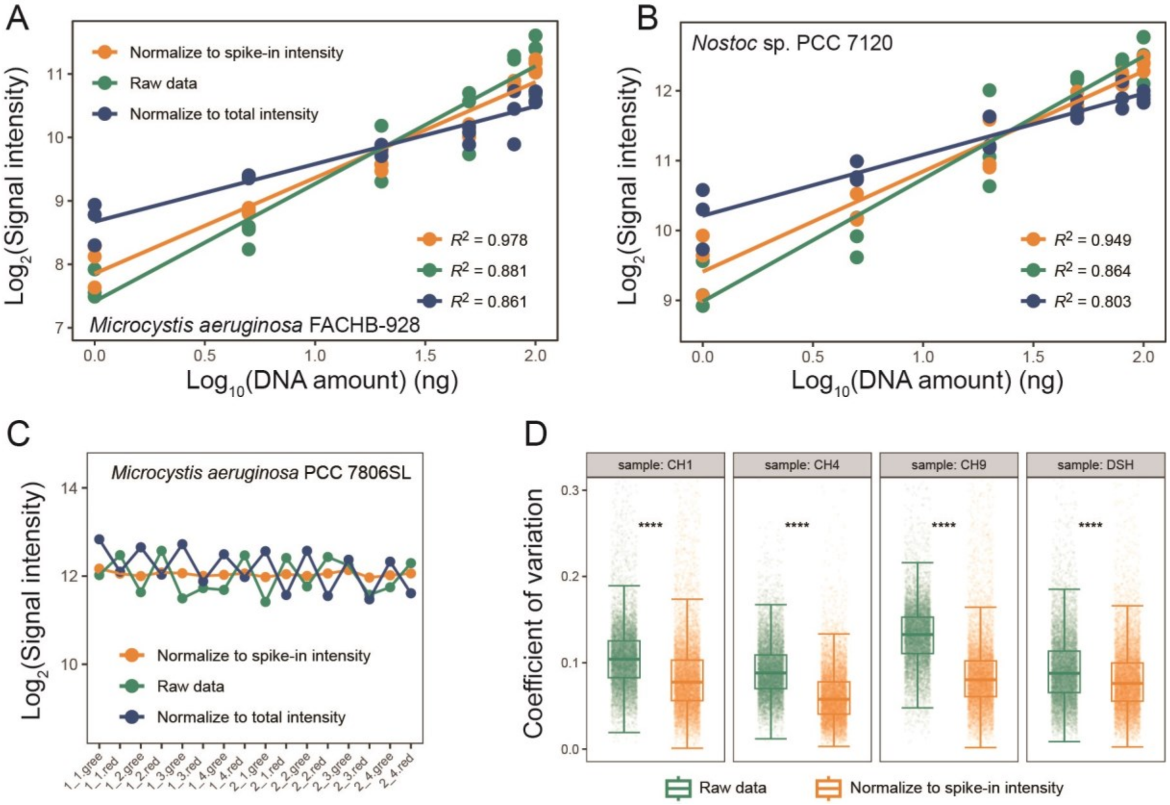

Figure S4. Utility of the spike-in control for CyanoStrainChip data normalization. Correlations between fluorescence intensities and DNA amounts for raw data, spike-in control normalized data, and global normalized data using *Microcystis aeruginosa* FACHB-928 (A) and *Nostoc* sp. PCC 7120 (B). (C) The fluctuation of strain PCC 7806SL signal intensities across 16 replicates for raw data, spike-in control normalized data, and global normalized data. (D) comparison of the variations of four field samples among inter-replicates between raw data and spike-in control normalized data. Comparisons were performed using a Mann–Whitney test (*, $P < 0.05$; **, $P < 0.01$; ***, $P < 0.001$; ****, $P < 0.0001$).

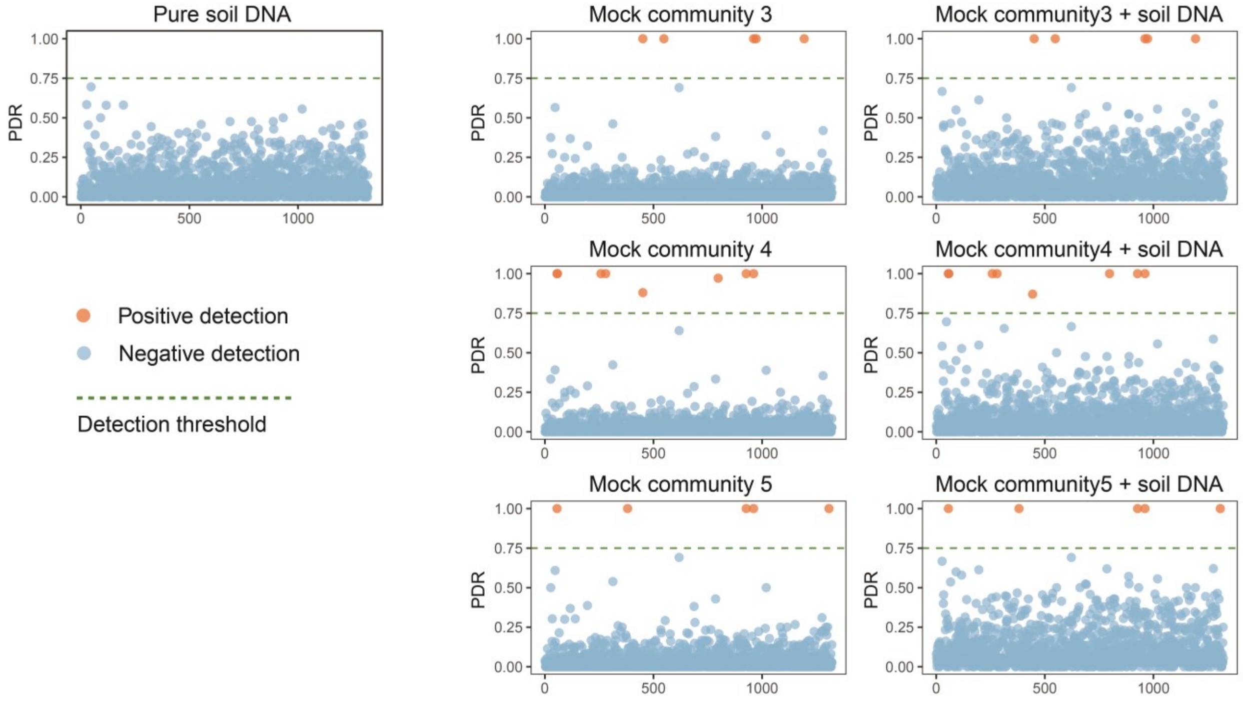

Figure S5. Evaluation of the effectiveness of CyanoStrainChip under the addition of a complex background DNA. The CyanoStrainChip detection outcomes of three type of samples: (1) pure urban grassland soil DNA, (2) mock communities comprised of pure strains, and (3) mock communities comprised of pure strains with urban grassland soil DNA as background were compared. All target strains arranged in a random order on the X-axis and the Y-axis represents corresponding Probe Detection Rate (PDR).

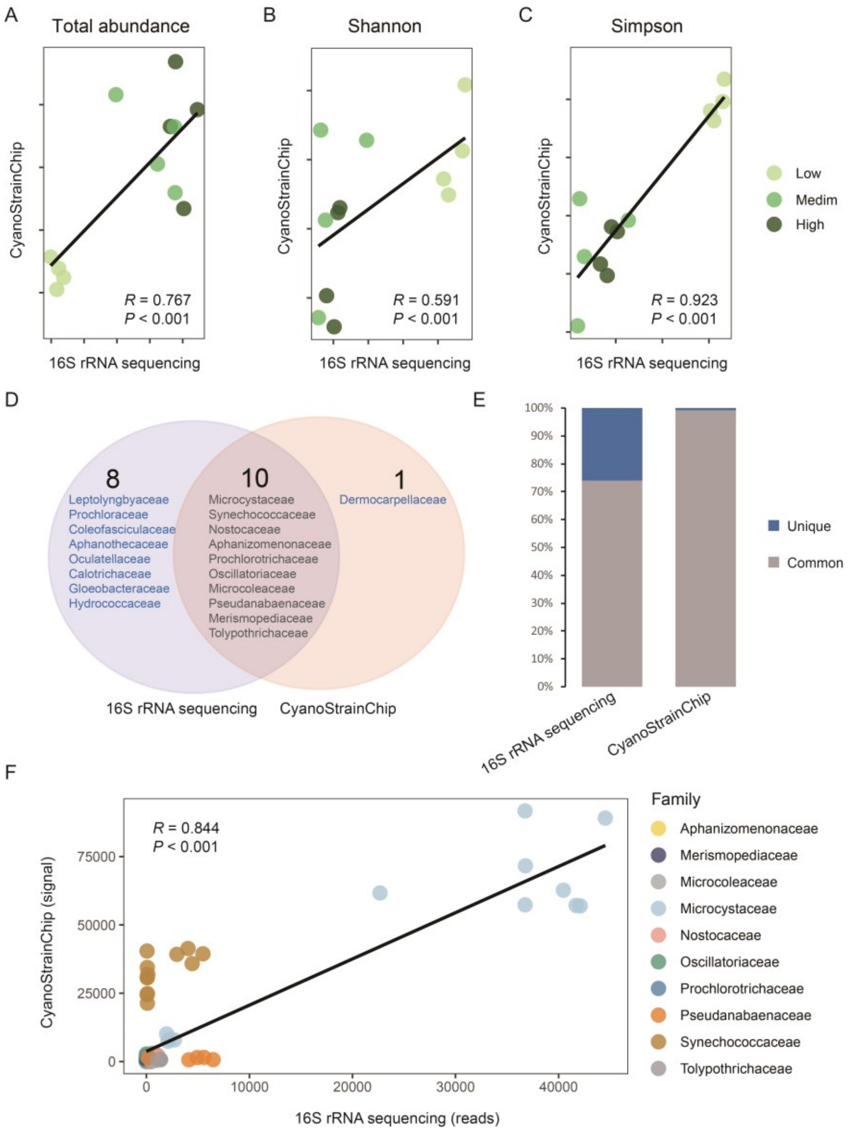

Figure S6. Comparison of CyanoStrainChip to 16S rRNA sequencing using water samples of cyanobacterial blooms from Chaohu. Correlation analysis of total abundance (A), Shannon index (B), and Simpson index (C) of the cyanobacterial community obtained from CyanoStrainChip and 16S rRNA sequencing. (D) Venn diagram of the cyanobacterial families uncovered by the two methods. (E) Proportional distribution of the relative abundance of unique and common detected families. (F) Scatterplot with abundance of cyanobacterial families detected in each replicate from the two methods.

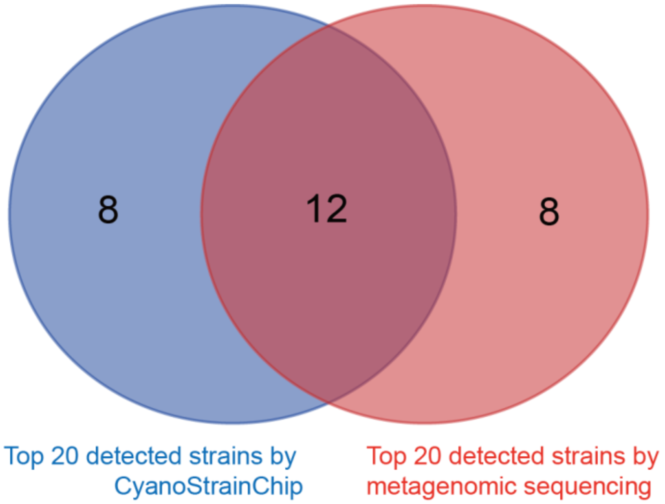

Figure S7. Venn diagram of the top 20 abundant cyanobacterial strains uncovered by CyanoStrainChip and metagenomic sequencing.

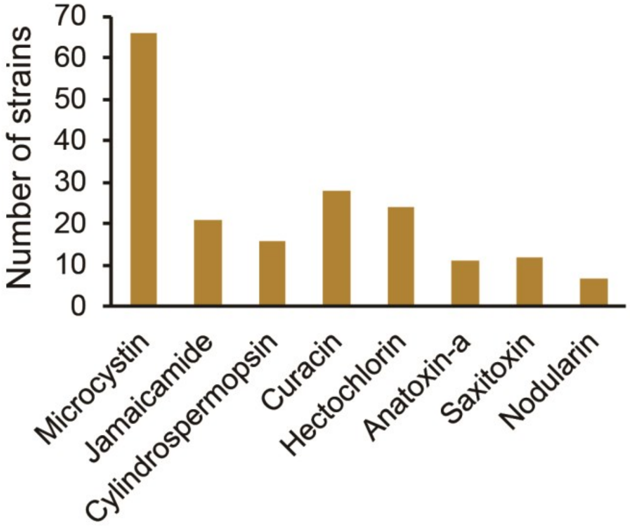

Figure S8. Statistical results of antiSMASH predictions of the toxin-producing features of all 1,277 cyanobacterial strains.

Table S2. Summary of used cyanobacterial strains in mock communities

Order	Genus	strain
Nostocales	Aphanizomenon	Aphanizomenon flos-aquae FACHB-1290
Nostocales	Aphanizomenon	Aphanizomenon flos-aquae FACHB-1416
Nostocales	Calothrix	Calothrix membranacea FACHB-236
Oscillatoriales	Desertifilum	Desertifilum sp. FACHB-866
Oscillatoriales	Limnospira	Limnospira indica PCC 8005
Synechococcales	Limnothrix	Limnothrix sp. FACHB-1083
Synechococcales	Limnothrix	Limnothrix sp. FACHB-1088
Chroococcales	Microcystis	Microcystis aeruginosa FACHB-524
Chroococcales	Microcystis	Microcystis aeruginosa FACHB-928
Chroococcales	Microcystis	Microcystis flos-aquae FACHB-1344
Chroococcales	Microcystis	Microcystis panniformis FACHB-1757
Chroococcales	Microcystis	Microcystis wesenbergii FACHB-1317
Nostocales	Nostoc	Nostoc flagelliforme FACHB-838
Nostocales	Nostoc	Nostoc foliaceum FACHB-393
Nostocales	Nostoc	Nostoc sp. FACHB-142
Nostocales	Nostoc	Nostoc sp. FACHB-973
Nostocales	Nostoc	Nostoc sp. PCC 7120 = FACHB-418
Oscillatoriales	Phormidium	Phormidium tenue FACHB-1052
Oscillatoriales	Phormidium	Phormidium tenue NIES-30
Oscillatoriales	Planktothrix	Planktothrix sp. FACHB-1365
Synechococcales	Pseudanabaena	Pseudanabaena sp. PCC 6802
Synechococcales	Synechococcus	Synechococcus elongatus PCC 6301
Synechococcales	Synechocystis	Synechocystis sp. PCC 6803

Table S3. Summary of strain-specific probes in CyanoStrainChip organized by main taxonomic groups

Order	Family	No. of Strains	No. of Probes
Chroococcales	Geminocystaceae	5	189
	Chroococcaceae	7	261
	Microcystaceae	58	1,939
	Aphanothecaceae	12	429
	Others	3	106
Chroococcidiopsidales	Chroococcidiopsidaceae	7	250
Gloeobacterales	Gloeobacteraceae	1	22
Gloeoemargaritales	Gloeomargaritaceae	1	25
Nostocales	Calotrichaceae	21	758
	Fortieaceae	3	107
	Tolypothrichaceae	4	146
	Aphanizomenonaceae	35	1,242
	Scytonemataceae	4	145
	Hapalosiphonaceae	22	725
	Nostocaceae	93	3,249
	Others	8	295
Oscillatoriales	Microcoleaceae	32	1,118
	Oscillatoriaceae	24	765
	Coleofasciculaceae	7	225
	Others	10	351
Pleurocapsales	Hyellaceae	5	188
	Others	2	70
Spirulinales	Spirulinaceae	2	62
Synechococcales	Acaryochloridaceae	3	96
	Oculatellaceae	2	60
	Merismopediaceae	7	210
	Leptolyngbyaceae	26	791
	Pseudanabaenaceae	23	784
	Prochloraceae	563	20,274
	Synechococcaceae	161	4,685
	Others	9	257
Unclassified	Unclassified	117	3,842
Sum		1,277	43,666

Table S4. R values for linear correlation of the signals of all specific probes to the total amounts of target DNA for *Microcystis aeruginosa* FACHB-928 and *Nostoc sp*. PCC7120

Strain	Probe name	R statistic	Significance
Nostoc sp. PCC 7120	cyo_2_1510	0.995	P < 0.001
Nostoc sp. PCC 7120	cyo_2_37869	0.998	P < 0.001
Nostoc sp. PCC 7120	cyo_2_4947	0.987	P < 0.001
Nostoc sp. PCC 7120	cyo_2_5137	0.999	P < 0.001
Nostoc sp. PCC 7120	cyo_2_22401	0.996	P < 0.001
Nostoc sp. PCC 7120	cyo_2_29511	0.995	P < 0.001
Nostoc sp. PCC 7120	cyo_2_50347	0.986	P < 0.001
Nostoc sp. PCC 7120	cyo_2_53618	0.981	P < 0.001
Nostoc sp. PCC 7120	cyo_2_7050	0.999	P < 0.001
Nostoc sp. PCC 7120	cyo_2_9730	0.980	P < 0.001
Nostoc sp. PCC 7120	cyo_2_51069	0.995	P < 0.001
Nostoc sp. PCC 7120	cyo_2_14707	0.999	P < 0.001
Nostoc sp. PCC 7120	cyo_2_13371	0.994	P < 0.001
Nostoc sp. PCC 7120	cyo_2_39095	0.984	P < 0.001
Nostoc sp. PCC 7120	cyo_2_8685	0.998	P < 0.001
Nostoc sp. PCC 7120	cyo_2_10194	0.989	P < 0.001
Nostoc sp. PCC 7120	cyo_2_47120	0.990	P < 0.001
Nostoc sp. PCC 7120	cyo_2_9522	0.995	P < 0.001
Nostoc sp. PCC 7120	cyo_2_16365	0.993	P < 0.001
Nostoc sp. PCC 7120	cyo_2_15646	0.991	P < 0.001
Nostoc sp. PCC 7120	cyo_2_47783	0.984	P < 0.001
Nostoc sp. PCC 7120	cyo_2_44292	0.990	P < 0.001
Nostoc sp. PCC 7120	cyo_2_1763	0.978	P < 0.001
Nostoc sp. PCC 7120	cyo_2_20365	0.991	P < 0.001
Nostoc sp. PCC 7120	cyo_2_22614	0.978	P < 0.001
Nostoc sp. PCC 7120	cyo_2_17580	0.982	P < 0.001
Nostoc sp. PCC 7120	cyo_2_19224	0.980	P < 0.001
Nostoc sp. PCC 7120	cyo_2_11243	0.969	P < 0.001
Nostoc sp. PCC 7120	cyo_2_54213	0.993	P < 0.001
Nostoc sp. PCC 7120	cyo_2_27961	0.979	P < 0.001
Microcystis aeruginosa FACHB-928	cyo_2_32803	0.997	P < 0.001
Microcystis aeruginosa FACHB-928	cyo_2_58057	0.999	P < 0.001
Microcystis aeruginosa FACHB-928	cyo_2_31712	0.997	P < 0.001
Microcystis aeruginosa FACHB-928	cyo_2_7884	0.998	P < 0.001
Microcystis aeruginosa FACHB-928	cyo_2_47492	0.999	P < 0.001
Microcystis aeruginosa FACHB-928	cyo_2_54269	0.999	P < 0.001
Microcystis aeruginosa FACHB-928	cyo_2_49407	0.999	P < 0.001
Microcystis aeruginosa FACHB-928	cyo_2_35725	1.000	P < 0.001
Microcystis aeruginosa FACHB-928	cyo_2_39152	1.000	P < 0.001
Microcystis aeruginosa FACHB-928	cyo_2_14000	1.000	P < 0.001
Microcystis aeruginosa FACHB-928	cyo_2_29929	0.999	P < 0.001

Microcystis aeruginosa FACHB-928	cyo_2_15650	1.000	P < 0.001
Microcystis aeruginosa FACHB-928	cyo_2_8935	0.999	P < 0.001
Microcystis aeruginosa FACHB-928	cyo_2_50646	0.999	P < 0.001
Microcystis aeruginosa FACHB-928	cyo_2_44525	1.000	P < 0.001
Microcystis aeruginosa FACHB-928	cyo_2_45096	0.999	P < 0.001
Microcystis aeruginosa FACHB-928	cyo_2_34890	0.999	P < 0.001
Microcystis aeruginosa FACHB-928	cyo_2_38463	0.999	P < 0.001
Microcystis aeruginosa FACHB-928	cyo_2_26022	0.999	P < 0.001
Microcystis aeruginosa FACHB-928	cyo_2_57090	1.000	P < 0.001
Microcystis aeruginosa FACHB-928	cyo_2_8235	0.999	P < 0.001
Microcystis aeruginosa FACHB-928	cyo_2_55800	0.999	P < 0.001
Microcystis aeruginosa FACHB-928	cyo_2_3787	1.000	P < 0.001
Microcystis aeruginosa FACHB-928	cyo_2_39470	0.999	P < 0.001
Microcystis aeruginosa FACHB-928	cyo_2_29157	0.999	P < 0.001
Microcystis aeruginosa FACHB-928	cyo_2_54145	0.996	P < 0.001
Microcystis aeruginosa FACHB-928	cyo_2_49710	1.000	P < 0.001
Microcystis aeruginosa FACHB-928	cyo_2_56402	0.996	P < 0.001
Microcystis aeruginosa FACHB-928	cyo_2_27093	0.998	P < 0.001
Microcystis aeruginosa FACHB-928	cyo_2_17535	0.998	P < 0.001
Microcystis aeruginosa FACHB-928	cyo_2_56619	0.996	P < 0.001
Microcystis aeruginosa FACHB-928	cyo_2_11721	0.998	P < 0.001
Microcystis aeruginosa FACHB-928	cyo_2_54555	0.999	P < 0.001
Microcystis aeruginosa FACHB-928	cyo_2_21761	0.999	P < 0.001
Microcystis aeruginosa FACHB-928	cyo_2_16290	0.998	P < 0.001
Microcystis aeruginosa FACHB-928	cyo_2_11602	0.998	P < 0.001

Table S5. The detected cyanobacteria profiles of cyanobacteria blooms at three sites in Lake Chaohu with third-level chlorophyll concentrations

Strain name	High .1	High .2	High .3	High .4	Medim .1	Medim .2	Medim .3	Medim .4	Low .1	Low .2	Low .3	Low .4	Order
Microcystis aeruginosa KW	7658	9382	11001	8920	8114	7562	7381	7223	507	561	890	592	Chroococcales
Microcystis aeruginosa NIES-843	7348	9304	8585	8330	10070	9654	10785	9276	520	764	817	774	Chroococcales
Microcystis aeruginosa PCC 9717	3486	4407	4525	3395	1683	1616	1731	1848	0	0	0	0	Chroococcales
Microcystis sp. M_QC_C_20170808_M9Col	3035	3425	3369	3511	576	549	619	595	0	0	0	0	Chroococcales
Microcystis wesenbergii FACHB-1317	2887	3488	3205	2584	2400	2055	2496	2411	381	0	0	378	Chroococcales
Microcystis ichthyoblabe FACHB-1294	2479	2678	2544	2163	1625	1631	1470	1520	0	783	0	781	Chroococcales
Microcystis wesenbergii FACHB-1336	2442	3640	3028	2089	4287	4251	4806	4312	593	586	502	386	Chroococcales
Microcystis sp. FACHB-1315	2066	2291	2245	2286	2010	2007	2023	1879	0	0	0	0	Chroococcales
Microcystis aeruginosa TA09	2014	2138	2143	1781	1654	1324	1577	1525	0	0	0	0	Chroococcales
Microcystis wesenbergii FACHB-1339	1947	2852	2347	2160	2118	2202	2598	2176	1036	1184	1043	1121	Chroococcales
Microcystis flos-aquae FACHB-1341	1932	2443	2407	2076	2227	1931	2150	2093	0	0	0	0	Chroococcales
Microcystis aeruginosa NIES-44	1890	2047	2332	1653	1081	1063	1172	1071	0	0	0	0	Chroococcales
Nostoc sp. 3335mG	1849	2101	2187	2175	2134	2305	2484	2446	1926	1925	2013	2196	Nostocales
Cyanobium sp. CACIAM 14	1668	1986	1678	1568	1866	2143	1868	1749	1773	1758	1625	1850	Synechococcales
Microcystis aeruginosa NIES-4285	1548	1986	1958	1755	765	795	856	883	0	0	0	0	Chroococcales
Microcystis flos-aquae FACHB-1330	1397	1622	1814	1417	1257	1235	1339	1438	0	0	0	0	Chroococcales
Microcystis aeruginosa NIES-2520	1370	1701	1994	1131	724	737	733	742	0	0	0	0	Chroococcales
Aphanizomenon flos-aquae FACHB-1416	1348	1465	1749	1852	1557	1637	2044	2158	2419	1912	1955	1761	Nostocales
Microcystis aeruginosa FACHB-524	1327	1336	1286	1433	338	438	433	462	0	0	0	0	Chroococcales
Synechococcus sp. RSCCF101	1307	1375	1508	1341	1419	1346	1530	1500	1339	1334	1273	1507	Synechococcales
Microcystis aeruginosa FACHB-1023	1304	1478	1329	1230	1404	1541	1429	1358	1399	1231	1400	1312	Chroococcales
cyanobacterium TDX16	1232	1128	1292	1371	0	1359	1662	1584	1486	1472	1454	1320	unclassified Cyanobacteria
Thermosynechococcus sp. NK55a	1169	0	1252	0	0	1183	1293	1211	1193	1199	1244	0	Synechococcales
Cyanobacteria bacterium QS_8_64_29	1133	1147	1073	1119	1255	1221	1168	1228	1322	1343	1271	1307	unclassified Cyanobacteria
Microcystis sp. M_OC_Ca_00000000_C217Col	1130	1191	1071	622	861	859	893	886	0	0	0	0	Chroococcales

Microcystis aeruginosa NIES-2549	1128	1429	1324	1268	867	887	838	871	0	0	0	0	Chroococcales
Microcystis flos-aquae FACHB-1323	1100	1186	1324	0	535	0	575	581	0	0	0	0	Chroococcales
Cyanobacteria bacterium T3Sed10_344R1	1090	1094	1216	1159	1135	1099	1244	1212	1108	1155	1165	1122	unclassified Cyanobacteria
Cyanobium sp. MED843	1030	933	1101	1111	1172	1029	1221	1196	1125	1134	1061	1089	Synechococcales
Cyanobacteria bacterium UBA999	993	1012	933	813	1009	1023	989	793	870	899	965	1004	unclassified Cyanobacteria
Microcystis aeruginosa FACHB-939	981	1349	1196	1313	0	1859	0	0	0	0	0	0	Chroococcales
Synechococcus sp. EAC657	963	767	1087	814	1063	793	1067	886	875	801	966	930	Synechococcales
Microcystis ichthyoblabe FACHB-1413	932	1084	1203	952	733	633	667	652	0	0	0	0	Chroococcales
Synechococcus sp. AG-670-B23	911	1045	0	0	910	0	936	0	0	0	991	0	Synechococcales
Microcystis elabens FACHB-917	899	972	981	0	989	1044	1093	0	0	0	0	1027	Chroococcales
Microcoleus sp. FACHB-1515	897	840	970	941	921	826	943	949	951	928	901	889	Chroococcales
Synechococcus sp. TMED90	869	0	882	844	949	0	957	920	895	942	878	941	Synechococcales
Synechococcus sp. MED850	856	860	990	922	869	928	936	983	964	997	979	1075	Synechococcales
Rubidibacter lacunae KORDI 51-2	846	895	833	738	887	878	870	820	822	796	786	802	Chroococcales
Cyanobacteria bacterium J007	839	969	861	893	835	864	820	843	878	873	850	893	unclassified Cyanobacteria
uncultured Synechococcus sp.	833	793	844	0	979	855	996	1032	954	977	964	870	Synechococcales
Synechococcus sp. AG-450-M17	833	827	795	827	0	855	813	0	831	826	837	953	Synechococcales
Cyanobium sp. PLM2.Bin73	829	876	926	842	904	936	945	916	894	902	867	946	Synechococcales
Limnothrix sp. CACIAM 69d	829	803	0	0	817	0	0	0	0	0	0	943	Synechococcales
Synechococcus sp. WH 5701	819	723	671	669	886	776	762	741	717	690	718	767	Synechococcales
Microcystis flos-aquae FACHB-1344	800	1011	1000	775	458	443	458	546	0	0	0	0	Chroococcales
Cyanobacteria bacterium T3Sed10_36R1	794	0	0	0	863	0	862	863	0	0	771	796	unclassified Cyanobacteria
Gloeobacter kilaueensis JS1	776	830	0	0	811	843	0	754	0	0	0	849	Gloeobacterales
Synechococcus sp. MED650	755	733	845	897	772	756	873	907	857	877	911	814	Synechococcales
Synechococcus sp. RS9917	739	750	835	794	799	764	873	750	702	738	726	719	Synechococcales
Microcystis panniformis Mp_MB_F_20080800_S26D	730	784	760	751	0	0	0	0	0	0	0	0	Chroococcales
Leptolyngbya sp. O-77	729	699	622	720	760	762	679	785	701	703	713	720	Synechococcales
Leptolyngbya sp. BC1307	729	739	655	671	803	782	703	688	767	772	711	794	Synechococcales
Synechococcus sp. RS9916	727	767	864	947	822	832	961	1047	945	939	942	941	Synechococcales
Microcystis sp. MC19	721	1066	1184	1040	0	0	0	739	0	0	0	0	Chroococcales

Synechococcus elongatus UTEX 3055	719	736	737	772	795	803	784	799	903	901	923	903	Synechococcales
Cyanobium gracile PCC 6307	710	771	763	887	705	735	828	0	853	862	809	752	Synechococcales
Geitlerinema sp. PCC 9228	687	693	758	0	695	0	754	699	731	715	710	739	Oscillatoriales
Cyanobacteria bacterium QS_5_48_63	684	675	737	0	741	734	756	783	723	738	711	772	unclassified Cyanobacteria
Microcystis sp. FACHB-1347	671	0	708	750	521	0	519	551	0	0	0	0	Chroococcales
Leptolyngbya sp. DLM2.Bin27	662	649	643	628	661	622	653	678	682	681	670	711	Synechococcales
Cyanobium sp. NIES-981	660	724	825	853	727	745	871	853	676	704	788	795	Synechococcales
Synechococcus sp. UW105	659	641	628	625	699	709	687	675	737	760	720	665	Synechococcales
Halomicronema hongdechloris C2206	653	559	618	634	722	615	652	711	659	648	635	627	Synechococcales
Limnothrix sp. FACHB-1088	641	722	688	649	673	698	725	660	633	667	655	647	Synechococcales
Cyanobacteria bacterium UBA5018	637	604	673	687	675	667	742	750	672	667	657	683	unclassified Cyanobacteria
Synechococcus sp. AG-683-F20	631	505	681	696	677	577	744	710	725	747	753	644	Synechococcales
Synechococcus sp. CC9605	627	0	0	0	0	0	0	0	0	0	0	781	Synechococcales
Synechococcus sp. SynAce01	621	593	585	587	663	586	602	579	598	615	553	588	Synechococcales
Leptolyngbya sp. DLM2.Bin15	586	604	674	746	615	662	733	758	699	699	666	709	Synechococcales
filamentous cyanobacterium CCP4	574	538	522	535	606	562	0	556	624	639	0	597	unclassified Cyanobacteria
Cyanobacteria bacterium J069	573	590	481	583	555	579	496	600	593	558	608	690	unclassified Cyanobacteria
Microcystis sp. M_OC_Ca_00000000_S217Cul	572	576	620	518	663	609	661	571	550	574	569	605	Chroococcales
Microcystis flos-aquae FACHB-1028	540	819	886	712	377	441	506	471	0	0	0	0	Chroococcales
Microcystis aeruginosa NIES-4264	539	739	791	543	325	340	329	357	0	0	0	0	Chroococcales
Cyanobacteria bacterium UBA947	531	522	550	530	536	511	524	533	533	510	533	522	unclassified Cyanobacteria
Synechococcus sp. ARS1019	529	585	560	494	566	579	595	523	587	627	610	681	Synechococcales
Microcystis aeruginosa NIES-298	525	662	0	700	0	0	831	0	0	0	0	0	Chroococcales
Candidatus Synechococcus spongiarum LMB bulk10D	509	492	546	0	536	497	530	543	514	519	545	492	Synechococcales
Microcystis aeruginosa CHAOHU 1326	495	889	731	759	758	713	959	675	0	0	0	0	Chroococcales
Synechococcus sp. CPC35	479	0	577	604	0	0	648	697	631	635	636	648	Synechococcales
Synechococcus sp. MIT S9508	477	499	487	498	540	523	505	546	562	573	552	527	Synechococcales
Phormidium sp. SL48-SHIP	474	0	0	470	485	506	0	0	0	0	513	0	Oscillatoriales
Leptolyngbyaceae cyanobacterium CSSed162cmB_428R1	440	442	460	477	0	482	494	505	487	486	482	498	Synechococcales

Synechococcus sp. TMED169	419	503	522	529	442	532	575	551	535	529	544	572	Synechococcales
Phormidesmis priestleyi	397	404	0	0	398	403	402	0	459	469	485	471	Synechococcales
Shackletoniella antarctica	394	420	387	427	454	490	466	480	435	462	461	502	Synechococcales
Leptolyngbya sp. 'hensonii'	360	372	439	435	0	376	412	430	360	364	371	391	Synechococcales
Lyngbya confervoides BDU141951	354	0	0	0	369	0	378	383	0	0	362	0	Oscillatoriales
Cyanobacteria bacterium B1Sed10_56	320	0	0	370	0	0	0	394	0	0	0	0	unclassified Cyanobacteria
Microcystis aeruginosa PCC 9809	313	396	367	332	279	294	277	303	0	0	0	0	Chroococcales
Aphanocapsa feldmannii 277cI	0	0	871	870	974	994	958	906	982	956	961	1067	Synechococcales
Aphanocapsa feldmannii 288cV	0	0	647	0	0	0	0	0	0	0	657	0	Synechococcales
Candidatus Synechococcus spongiarum SH4	0	0	524	551	0	0	578	637	550	555	553	591	Synechococcales
Candidatus Synechococcus spongiarum LMB bulk10E	0	0	0	0	677	743	0	717	547	564	544	562	Synechococcales
Candidatus Synechococcus spongiarum LMB bulk15N	0	407	503	505	0	413	513	530	539	561	545	502	Synechococcales
Candidatus Synechococcus spongiarum	0	1046	1037	962	0	1141	1048	1025	959	1094	1092	1039	Synechococcales
Hydrococcus rivularis NIES-593	0	0	0	0	0	0	716	688	0	0	0	0	Pleurocapsales
Leptolyngbya foveolarum	0	645	566	618	0	721	654	631	680	644	0	716	Synechococcales
Leptolyngbya ohadii IS1	0	0	922	916	991	0	991	949	936	943	0	955	Synechococcales
Microcystis aeruginosa NIES-88	0	572	593	489	0	0	0	0	0	0	0	0	Chroococcales
Microcystis aeruginosa CACIAM 03	0	318	0	350	331	341	0	364	403	381	370	345	Chroococcales
Microcystis aeruginosa 11-30S32	0	4793	5112	3517	0	0	0	0	0	0	0	0	Chroococcales
Microcystis aeruginosa EAWAG127a	0	0	1021	0	0	0	969	880	0	0	0	0	Chroococcales
Microcystis aeruginosa FACHB-1279	0	781	903	866	784	900	1046	948	864	947	900	947	Chroococcales
Microcystis flos-aquae FACHB-1332	0	5018	5089	0	2046	1738	1639	1922	0	0	0	0	Chroococcales
Microcystis aeruginosa FACHB-978	0	0	1275	1080	0	663	851	876	0	0	0	0	Chroococcales
Microcystis panniformis FACHB-1757	0	1927	1966	1603	0	0	0	0	0	0	0	0	Chroococcales
Neosynechococcus sphagnicola sy1	0	0	515	0	0	0	549	0	0	495	505	488	Synechococcales
Nodosilinea nodulosa PCC 7104	0	0	0	0	0	0	0	0	630	667	654	608	Synechococcales
Synechococcus lacustris	0	0	0	0	0	517	0	0	0	529	536	524	Synechococcales
Thermosynechococcus elongatus BP-1	0	594	0	0	0	649	743	0	0	0	0	0	Synechococcales
Tolypothrix campylonemoides VB511288	0	0	0	708	0	0	754	713	704	705	716	637	Nostocales
Cyanobacteria bacterium TMED177	0	0	1337	1384	0	0	1349	1428	1138	1203	1232	1057	unclassified Cyanobacteria
Cyanobacteria bacterium SW_6_48_11	0	648	668	625	735	710	772	693	719	692	696	726	unclassified

													Cyanobacteria
Cyanobacteria bacterium SW_9_44_58	0	0	0	0	0	0	0	0	0	0	450	475	unclassified Cyanobacteria
filamentous cyanobacterium CCP3	0	0	527	0	0	0	0	0	0	0	0	555	unclassified Cyanobacteria
Cyanobacteria bacterium UBA11368	0	0	0	0	0	0	1119	1031	0	0	0	0	unclassified Cyanobacteria
Synechococcales bacterium UBA8138	0	0	0	0	0	0	0	0	0	0	774	689	Synechococcales
Cyanobacteria bacterium CSSed11_75	0	0	0	485	0	0	0	0	593	600	592	0	unclassified Cyanobacteria
Cyanobacteria bacterium J055	0	669	692	662	0	704	765	735	718	675	688	681	unclassified Cyanobacteria
Calothrix sp. FACHB-1219	0	0	0	0	631	624	718	682	584	623	602	553	Nostocales
Cyanobium sp. ARS6	0	0	522	559	0	0	0	567	554	0	519	0	Synechococcales
Geitlerinema sp. PCC 7407	0	0	0	0	882	972	0	0	973	1080	1104	1023	Oscillatoriales
Geitlerinema sp. BBD	0	481	493	531	568	518	549	600	552	529	543	525	Oscillatoriales
Leptolyngbya sp. PCC 6406	0	0	743	603	0	689	753	619	646	652	674	714	Synechococcales
Leptolyngbya sp. IPPAS B-1204	0	0	0	0	0	404	0	0	0	0	0	385	Synechococcales
Microcystis sp. Msp_OC_L_20101000_S702	0	0	0	0	0	1071	1064	0	1026	0	0	966	Chroococcales
Nodosilinea sp. CSSed162cmB_84	0	0	468	502	0	588	559	598	604	572	560	623	Synechococcales
Oscillatoria sp. PCC 10802	0	0	657	0	0	0	670	0	0	0	0	0	Oscillatoriales
Oscillatoria sp. FACHB-1406	0	0	0	860	0	0	0	902	869	876	893	945	Oscillatoriales
Phormidium sp.	0	0	0	478	0	0	432	0	0	0	494	506	Oscillatoriales
Phormidium sp. GEM2.Bin31	0	0	0	0	0	0	0	0	504	492	497	536	Oscillatoriales
Planktothricoides sp. SR001	0	0	0	0	513	526	560	555	0	0	0	0	Oscillatoriales
Planktothricoides sp. FACHB-1261	0	0	0	0	1378	1425	1498	1427	0	0	0	0	Oscillatoriales
Planktothricoides sp. FACHB-1370	0	0	0	0	907	976	998	963	401	381	399	393	Oscillatoriales
Synechococcus sp. JA-2-3B'a(2-13)	0	0	580	585	0	0	567	571	557	557	524	599	Synechococcales
Synechococcus sp. WH 7805	0	0	353	403	344	349	362	394	393	392	380	357	Synechococcales
Synechococcus sp. BL107	0	0	0	0	706	0	687	655	0	589	599	618	Synechococcales
Synechococcus sp. PCC 7336	0	0	0	0	0	0	927	0	0	892	871	875	Synechococcales
Synechococcus sp. KORDI-100	0	467	0	0	0	522	546	0	526	513	505	528	Synechococcales
Synechococcus sp. KORDI-52	0	0	698	758	0	751	706	774	806	818	742	803	Synechococcales
Synechococcus sp. WH 8020	0	0	0	0	0	0	578	556	0	604	0	0	Synechococcales
Synechococcus sp. REDSEA-S01_B1	0	0	0	706	0	0	0	0	670	668	641	681	Synechococcales

Synechococcus sp. REDSEA-S02_B4	0	685	605	651	0	734	654	712	736	680	695	673	Synechococcales
Synechococcus sp. TMED19	0	742	0	695	0	742	646	673	682	681	661	707	Synechococcales
Synechococcus sp. TMED155	0	596	0	709	0	0	657	723	700	700	713	706	Synechococcales
Synechococcus sp. NAT40	0	0	0	0	0	0	383	0	398	387	398	406	Synechococcales
Synechococcus sp. 63AY4M2	0	567	516	488	559	562	522	524	538	558	569	599	Synechococcales
Synechococcus sp. AG-683-G11	0	0	0	0	0	0	708	0	0	0	695	0	Synechococcales
Synechococcus sp. AG-679-A04	0	0	636	0	487	664	703	697	644	641	649	654	Synechococcales
Synechococcus sp. AG-676-E23	0	0	735	738	0	739	763	803	757	708	731	753	Synechococcales
Synechococcus sp. AG-673-F03	0	522	0	535	594	545	577	564	638	619	637	592	Synechococcales
Synechococcus sp. AG-673-A03	0	0	0	0	0	0	0	0	0	661	643	558	Synechococcales
Synechococcus sp. MED-G71	0	0	730	731	848	778	795	789	806	766	807	805	Synechococcales
Synechococcus sp. MED-G67	0	788	912	829	0	777	825	910	772	740	742	656	Synechococcales
Synechococcus sp. UBA8638	0	0	541	534	0	0	592	0	521	521	528	583	Synechococcales
Synechococcus sp. BS56D	0	0	553	518	0	673	579	552	604	641	574	617	Synechococcales
Synechococcus sp. BS55D	0	0	562	564	496	0	617	0	577	597	586	555	Synechococcales
Synechococcus sp. N32	0	0	0	528	0	0	0	0	0	602	593	644	Synechococcales
Synechococcus sp. UW140	0	0	724	0	0	0	770	750	0	0	675	610	Synechococcales

Tables S6. The detected cyanobacteria profiles of one water sample from Reservoir Dashahe by the CyanoStrainChip and metagenomic short sequencing

Strain name	Assembly	Completeness	PDR	Depth (Log10)	Signal intensity (Log2)	Family
Cylindrospermopsis raciborskii GIHE 2018	GCA_006523545.1	0.90	0.97	4.01	11.44	Aphanizomenonaceae
Cylindrospermopsis sp. CR12	GCA_001432185.1	0.89	1.00	3.86	13.07	Aphanizomenonaceae
Limnothrix sp. FACHB-1083	GCA_014696485.1	0.65	0.96	3.02	10.01	Pseudanabaenaceae
Synechococcus sp. RSCCF101	GCA_008807075.1	0.62	0.90	2.45	10.48	Synechococcaceae
Nostoc sp. 3335mG	GCA_003185865.1	0.58	1.00	2.26	11.10	Nostocaceae
Limnothrix sp. FACHB-1088	GCA_014696455.1	0.53	0.94	2.52	9.33	Pseudanabaenaceae
Cylindrospermopsis raciborskii S01	GCA_002893285.1	0.53	0.76	3.35	9.87	Aphanizomenonaceae
cyanobacterium TDX16	GCA_002213405.1	0.41	0.76	2.18	10.32	unclassified
Synechococcus sp. SynAce01	GCA_001885215.1	0.40	0.77	2.62	9.19	Synechococcaceae
Microcystis aeruginosa EAWAG127a	GCA_008757435.1	0.39	0.76	2.10	9.59	Microcystaceae
Cyanobacteria bacterium QS_8_64_29	GCA_003022125.1	0.38	0.96	2.24	10.19	unclassified
Synechococcus sp. ARS1019	GCA_002690325.1	0.34	0.79	2.20	9.32	Synechococcaceae
Cyanobium sp. CACIAM 14	GCA_000708525.1	0.33	0.78	2.04	10.99	Synechococcaceae
Synechococcus sp. CPC35	GCA_002684175.1	0.32	0.82	1.93	9.24	Synechococcaceae
Gloeobacter kilaueensis JS1	GCA_000484535.1	0.32	0.77	1.76	9.82	Gloeobacteraceae
Candidatus Synechococcus spongiarum	GCA_900047545.1	0.32	0.77	2.20	9.98	Synechococcaceae

Synechococcus sp. AG-683-F20	GCA_003210495.1	0.28	0.80	2.06	9.16	Synechococcaceae
Cyanobium sp. NIES-981	GCA_900088535.1	0.28	0.76	1.64	9.47	Synechococcaceae
Synechococcus sp. RS9917	GCA_000153065.1	0.28	0.83	1.69	9.45	Synechococcaceae
Synechococcus sp. MED-G67	GCA_003331795.1	0.27	0.83	2.34	9.36	Synechococcaceae
Cyanobacteria bacterium UBA5018	GCA_002396505.1	0.27	0.76	2.25	9.47	unclassified
Cyanobacteria bacterium QS_5_48_63	GCA_003022205.1	0.26	0.80	2.73	9.39	unclassified
Cyanobacteria bacterium TMED177	GCA_002170825.1	0.22	0.80	2.29	10.08	unclassified
Cyanobacteria bacterium SW_6_48_11	GCA_003021845.1	0.22	0.75	2.71	9.25	unclassified
Leptolyngbya sp. DLM2.Bin15	GCA_007694125.1	0.21	0.79	1.71	9.50	Leptolyngbyaceae
Synechococcus sp. TMED19	GCA_002168155.1	0.21	0.75	1.80	9.58	Synechococcaceae
Synechococcus sp. 63AY4M2	GCA_002760475.1	0.21	0.81	2.27	9.26	Synechococcaceae
Aphanizomenon flos-aquae FACHB-1416	GCA_014698695.1	0.20	0.91	2.23	10.77	Aphanizomenonaceae
Leptolyngbya sp. PCC 6406	GCA_000332095.2	0.19	0.77	1.70	9.72	Leptolyngbyaceae
Synechococcus sp. AG-679-A04	GCA_003210775.1	0.18	0.75	2.04	9.37	Synechococcaceae
Microcystis aeruginosa KW	GCA_002025445.1	0.17	0.76	1.61	8.67	Microcystaceae
Cyanobium sp. PLM2.Bin73	GCA_007694915.1	0.17	0.83	1.42	9.83	Synechococcaceae
Leptolyngbya sp. DLM2.Bin27	GCA_007694115.1	0.17	0.75	2.08	9.49	Leptolyngbyaceae
Shackletoniella antarctica	GCA_003241845.1	0.16	0.80	1.89	9.06	Oculatellaceae
Halomicronema hongdechloris C2206	GCA_002075285.3	0.15	0.81	1.85	9.20	Prochlorotrichaceae
Candidatus Synechococcus spongiarum LMB	GCA_002017955.1	0.15	0.75	1.55	9.35	Synechococcaceae

bulk10E						
Synechococcus sp. MED850	GCA_002700765.1	0.14	0.79	2.70	9.89	Synechococcaceae
Synechococcus sp. TMED169	GCA_002172175.1	0.14	0.79	1.39	9.13	Synechococcaceae
Synechococcus sp. REDSEA-S02_B4	GCA_001628325.1	0.14	0.77	1.59	9.32	Synechococcaceae
Synechococcus sp. KORDI-52	GCA_000737595.1	0.14	0.75	1.49	9.56	Synechococcaceae
Synechococcus sp. AG-676-E23	GCA_003210935.1	0.14	0.75	1.56	9.45	Synechococcaceae
Cyanobacteria bacterium J069	GCA_003695655.1	0.13	0.83	1.56	9.33	unclassified
Synechococcus sp. WH 5701	GCA_000153045.1	0.13	0.88	1.41	9.61	Synechococcaceae
Synechococcus sp. MED650	GCA_002691345.1	0.12	0.79	2.23	9.40	Synechococcaceae
Synechococcus sp. WH 7805	GCA_000153285.1	0.12	0.77	1.27	8.49	Synechococcaceae
Synechococcus sp. MIT S9508	GCA_001632165.1	0.11	0.75	1.24	8.97	Synechococcaceae
Leptolyngbya sp. 'hensonii'	GCA_001939115.1	0.11	0.75	1.22	8.53	Leptolyngbyaceae
Cyanobacteria bacterium UBA947	GCA_002293275.1	0.09	0.76	1.97	8.98	unclassified
Prochlorococcus sp. UBA7854	GCA_002500205.1	0.09	0.81	1.61	9.30	Prochloraceae
Leptolyngbya sp. BC1307	GCA_002286735.1	0.07	0.76	0.80	9.63	Leptolyngbyaceae
Synechococcus sp. AG-673-F03	GCA_003211205.1	0.06	0.77	2.27	9.17	Synechococcaceae
Synechococcus sp. RS9916	GCA_000153825.1	0.05	0.76	0.60	9.80	Synechococcaceae

Table S7. Summary of advantages and disadvantages of methods for detection of environmental cyanobacteria

Method	Advantages	Disadvantages	References
16S rRNA sequencing	High-throughput (recover majority of cyanobacteria); Well-standardised; Multiple samples can be examined at a time; low cost	PCR amplification is needed; Cannot reach species-level due to the limitation of sequencing length; Complex analysis; Limited quantitative efficacy	Nübel, Garcia-Pichel, and Muyzer, 1997
Metagenomic sequencing	High-throughput (recover majority of cyanobacteria); Strain-level taxonomic resolution; No PCR amplification; Able to provide near-complete genomic sequence	High cost; Complex analysis; Massive computing power is required; Low reproducibility; Limited quantitative efficacy	Steffen et al., 2012; Alvarenga et al., 2017
Functional amplicon sequencing (eg., mcyB)	High specificity, suitable for detection of function-specific group; Multiple samples can be examined at a time; low cost; Able to provide gene sequence	PCR amplification is needed; Cannot reach species-level due to the limitation of sequencing length; Complex analysis; Limited quantitative efficacy; Sensitive to primer design	Casero et al., 2019; Padovan et al., 2023
Real-time PCR	High sensitivity; High specificity, suitable for detection of function-specific group; High quantitative efficacy	Require expensive equipment; Sensitive to primer design; Do not provide sequence information	Al-Tebrineh et al., 2012; Lu et al., 2020
Antibody microarray	High sensitivity; High specificity, suitable for detection of function-specific group or specific strains; Supports in situ testing	Cannot discover off-target cyanobacteria; May involve cross-reactions; low-throughput (detecting several to dozens of strains at a time)	Blanco et al., 2015
CyanoStrainChip	High-throughput (detecting thousands strains at a time); High specificity; Strain-level taxonomic resolution; High quantitative efficacy; High reproducibility; No PCR amplification	Cannot discover off-target cyanobacteria; May involve nonspecific hybridization; Require expensive equipment	This study
16S rRNA target DNA microarray	High-throughput (recover majority of cyanobacteria); High quantitative efficacy; High reproducibility;	PCR amplification is needed; May involve nonspecific hybridization; Require expensive equipment; Cannot reach species-level due to the limitation of sequencing length	Castiglioni et al., 2004
Culture + WGS	Able to recognize viable cells in a sample; High sensitivity and specificity with appropriate media; Able to provide comprehensive genomic information; Assesses culturable cyanobacteria	Time-consuming; low-throughput; many cyanobacteria are uncultured	Nakayama et al., 2014; Tang et al., 2019
Duplex dPCR	High sensitivity; High specificity, suitable for detection of function-specific group; High quantitative efficacy; Low cost; High tolerance for PCR inhibitor	Sensitive to primer design; Do not provide taxonomic information	Tan et al., 2021

REFERENCES

Al-Tebrineh, J., Pearson, L. A., Yasar, S. A., & Neilan, B. A. (2012). A multiplex qPCR targeting hepato-and neurotoxigenic cyanobacteria of global significance. Harmful Algae, 15, 19-25.

Alvarenga, D. O., Fiore, M. F., & Varani, A. M. (2017). A metagenomic approach to cyanobacterial genomics. Frontiers in microbiology, 8, 809.

Blanco, Y., Quesada, A., Gallardo-Carreño, I., Aguirre, J., & Parro, V. (2015). CYANOCHIP: an antibody microarray for high-taxonomical-resolution cyanobacterial monitoring. Environmental Science & Technology, 49(3), 1611-1620.

Blin, K., Shaw, S., Steinke, K., Villebro, R., Ziemert, N., Lee, S. Y., ... & Weber, T. (2019). antiSMASH 5.0: updates to the secondary metabolite genome mining pipeline. Nucleic acids research, 47(W1), W81-W87.

Buchfink, B., Xie, C., & Huson, D. H. (2015). Fast and sensitive protein alignment using DIAMOND. Nature methods, 12(1), 59-60.

Caporaso, J. G., Kuczynski, J., Stombaugh, J., Bittinger, K., Bushman, F. D., Costello, E. K., ... & Knight, R. (2010). QIIME allows analysis of high-throughput community sequencing data. Nature methods, 7(5), 335-336.

Casero, M. C., Velázquez, D., Medina-Cobo, M., Quesada, A., & Cirés, S. (2019). Unmasking the identity of toxigenic cyanobacteria driving a multi-toxin bloom by high-throughput sequencing of cyanotoxins genes and 16S rRNA metabarcoding. Science of the Total Environment, 665, 367-378.

Castiglioni, B., Rizzi, E., Frosini, A., Sivonen, K., Rajaniemi, P., Rantala, A., ... & De Bellis, G. (2004). Development of a universal microarray based on the ligation detection reaction and 16S rRNA gene polymorphism to target diversity of cyanobacteria. Applied and Environmental Microbiology, 70(12), 7161-7172.

Chen, K., Hu, Z., Xia, Z., Zhao, D., Li, W., & Tyler, J. K. (2015). The overlooked fact: fundamental need for spike-in control for virtually all genome-wide analyses. Molecular and cellular biology.

Edgar, R. C. (2013). UPARSE: highly accurate OTU sequences from microbial amplicon reads. Nature methods, 10(10), 996-998.

Gautier, L., Cope, L., Bolstad, B. M., & Irizarry, R. A. (2004). affy—analysis of Affymetrix GeneChip data at the probe level. Bioinformatics, 20(3), 307-315.

Grau, J., Grosse, I., & Keilwagen, J. (2015). PRROC: computing and visualizing precision-recall and receiver operating characteristic curves in R. Bioinformatics, 31(15), 2595-2597.

Hyatt, D., Chen, G. L., LoCascio, P. F., Land, M. L., Larimer, F. W., & Hauser, L. J. (2010). Prodigal: prokaryotic gene recognition and translation initiation site identification. BMC bioinformatics, 11, 1-11.

Kalantar, K. L., Carvalho, T., de Bourcy, C. F., Dimitrov, B., Dingle, G., Egger, R., ... & DeRisi, J. L. (2020). IDseq—An open source cloud-based pipeline and analysis service for metagenomic pathogen detection and monitoring. Gigascience, 9(10), giaa111.

Kane, M. D., Jatkoe, T. A., Stumpf, C. R., Lu, J., Thomas, J. D., & Madore, S. J. (2000). Assessment of the sensitivity and specificity of oligonucleotide (50mer) microarrays. Nucleic acids research, 28(22), 4552-4557.

Kautsar, S. A., Blin, K., Shaw, S., Navarro-Muñoz, J. C., Terlouw, B. R., Van Der Hooft, J. J., ... & Medema, M. H. (2020). MIBiG 2.0: a repository for biosynthetic gene clusters of known function. Nucleic acids research, 48(D1), D454-D458.

Langmead, B., & Salzberg, S. L. (2012). Fast gapped-read alignment with Bowtie 2. Nature methods, 9(4), 357-359.

Li, X., Harwood, V.J., Nayak, B., Staley, C., Sadowsky, M.J. and Weidhaas, J., 2015. A novel microbial source tracking microarray for pathogen detection and fecal source identification in environmental systems. Environmental science & technology, 49(12), pp.7319-7329.

Li, X., He, Z., & Zhou, J. (2005). Selection of optimal oligonucleotide probes for microarrays using multiple criteria, global alignment and parameter estimation. Nucleic acids research, 33(19), 6114-6123.

Liang, Y., He, Z., Wu, L., Deng, Y., Li, G., & Zhou, J. (2010). Development of a common oligonucleotide reference standard for microarray data normalization and comparison across different microbial communities. Applied and environmental microbiology, 76(4), 1088-1094.

Lu, J., Struewing, I., Wymer, L., Tettenhorst, D. R., Shoemaker, J., & Allen, J. (2020). Use of qPCR and RT-qPCR for monitoring variations of microcystin producers and as an early warning system to predict toxin production in an Ohio inland lake. Water research, 170, 115262.

Marçais, G., & Kingsford, C. (2011). A fast, lock-free approach for efficient parallel counting of occurrences of k-mers. Bioinformatics, 27(6), 764-770.

Nakayama, T., Kamikawa, R., Tanifuji, G., Kashiyama, Y., Ohkouchi, N., Archibald, J. M., & Inagaki, Y. (2014). Complete genome of a nonphotosynthetic cyanobacterium in a diatom reveals recent adaptations to an intracellular lifestyle. Proceedings of the National Academy of Sciences, 111(31), 11407-11412.

Nübel, U., Garcia-Pichel, F., & Muyzer, G. (1997). PCR primers to amplify 16S rRNA genes from cyanobacteria. Applied and environmental microbiology, 63(8), 3327-3332.

Padovan, A., Kennedy, K., & Gibb, K. (2023). A microcystin synthesis mcyE/ndaF gene assay enables early detection of microcystin production in a tropical wastewater pond. Harmful Algae, 127, 102476.

Schloss, P. D., Westcott, S. L., Ryabin, T., Hall, J. R., Hartmann, M., Hollister, E. B., ... & Weber, C. F. (2009). Introducing mothur: open-source, platform-independent, community-supported software for describing and comparing microbial communities. Applied and environmental microbiology, 75(23), 7537-7541.

Shi, Z., Yin, H., Van Nostrand, J.D., Voordeckers, J.W., Tu, Q., Deng, Y., Yuan, M., Zhou, A., Zhang, P., Xiao, N. and Ning, D., 2019. Functional gene array-based ultrasensitive and quantitative detection of microbial populations in complex communities. MSystems, 4(4), pp.e00296-19.

Steffen, M. M., Li, Z., Effler, T. C., Hauser, L. J., Boyer, G. L., & Wilhelm, S. W. (2012). Comparative metagenomics of toxic freshwater cyanobacteria bloom communities on two continents.

Tan, F., Xiao, P., Yang, J. R., Chen, H., Jin, L., Yang, Y., ... & Yang, J. (2021). Precision early detection of invasive and toxic cyanobacteria: a case study of Raphidiopsis raciborskii. Harmful Algae, 110, 102125.

Tang, J., Du, L. M., Liang, Y. M., & Daroch, M. (2019). Complete genome sequence and comparative analysis of Synechococcus sp. CS-601 (SynAce01), a cold-adapted cyanobacterium from an oligotrophic Antarctic habitat. International Journal of Molecular Sciences, 20(1), 152.

Tourlousse, D. M., Yoshiike, S., Ohashi, A., Matsukura, S., Noda, N., & Sekiguchi, Y. (2017). Synthetic spike-in standards for high-throughput 16S rRNA gene amplicon sequencing. Nucleic Acids Research, 45(4), e23-e23.

Tu, Q., He, Z., & Zhou, J. (2014). Strain/species identification in metagenomes using genome-specific markers. Nucleic acids research, 42(8), e67-e67.

Zahurak, M., Parmigiani, G., Yu, W., Scharpf, R. B., Berman, D., Schaeffer, E., ... & Cope, L. (2007). Pre-processing Agilent microarray data. BMC bioinformatics, 8, 1-13.
